# Ccr4-not ubiquitin ligase signaling regulates ribosomal protein homeostasis and inhibits 40S ribosomal autophagy

**DOI:** 10.1016/j.jbc.2024.107582

**Published:** 2024-07-16

**Authors:** Daniel L. Johnson, Ravinder Kumar, David Kakhniashvili, Lawrence M. Pfeffer, R. Nicholas Laribee

**Affiliations:** 1 Molecular Bioinformatics Core and the University of Tennessee Health Science Center Office of Research, University of Tennessee Health Science Center, Memphis, Tennessee, USA; 2 Department of Pathology and Laboratory Medicine, College of Medicine and the Center for Cancer Research, University of Tennessee Health Science Center, Memphis, Tennessee, USA; 3 Proteomics and Metabolomics Core and the University of Tennessee Health Science Center Office of Research, University of Tennessee Health Science Center, Memphis, Tennessee, USA

**Keywords:** Ccr4-not, ubiquitin ligase, ribosome, autophagy, microautophagy, lysosome

## Abstract

The Ccr4-Not complex contains the poorly understood Not4 ubiquitin ligase that functions in transcription, mRNA decay, translation, proteostasis, and endolysosomal nutrient signaling. To gain further insight into the *in vivo* functions of the ligase, we performed quantitative proteomics in *Saccharomyces cerevisiae* using yeast cells lacking Not4, or cells overexpressing wild-type Not4 or an inactive Not4 mutant. Herein, we provide evidence that balanced Not4 activity maintains ribosomal protein (RP) homeostasis independent of changes to RP mRNA or known Not4 ribosomal substrates. Intriguingly, we also find that Not4 loss activates 40S ribosomal autophagy independently of canonical Atg7-dependent macroautophagy, indicating that microautophagy is responsible. We previously demonstrated that Ccr4-Not stimulates the target of rapamycin complex 1 (TORC1) signaling, which activates RP expression and inhibits autophagy, by maintaining vacuole V-ATPase H^+^ pump activity. Importantly, combining Not4 deficient cells with a mutant that blocks vacuole H^+^ export fully restores RP expression and increases 40S RP autophagy efficiency. In contrast, restoring TORC1 activity alone fails to rescue either process, indicating that Not4 loss disrupts additional endolysosomal functions that regulate RP expression and 40S autophagy. Analysis of the Not4-regulated proteome reveals increases in endolysosomal and autophagy-related factors that functionally interact with Not4 to control RP expression and affect 40S autophagy. Collectively, our data indicate that balanced Ccr4-Not ubiquitin ligase signaling maintains RP homeostasis and inhibits 40S autophagy *via* the ligase’s emerging role as an endolysosomal regulator.

Cells dynamically respond to nutrient availability and stress through mechanisms that require communication between the gene expression machinery and metabolic-responsive organelles, including the endolysosomal compartment and mitochondria (1, 2). This communication allows cells to coordinate their growth and proliferation with favorable nutrient environments, while it has an equally significant role in limiting anabolic and proliferative activity under unfavorable environments. The dysregulation of these processes causes many complex diseases and developmental syndromes, including cancer, neurodevelopmental disorders, aging, and metabolic disease (2). Deciphering these interrelationships remains an unrealized goal as they are inherently complex with considerable functional redundancy.

The Ccr4-Not complex is a large multisubunit complex that is conserved throughout evolution and has critical roles throughout the gene expression process. Ccr4-Not originally was identified in budding yeast where it consists of the Ccr4, Caf1, Caf40, Caf130, and the Not1-5 subunits (3). The complex’s best-understood role is the regulation of cytoplasmic mRNA decay *via* the Ccr4 deadenylase subunit, and Ccr4-Not mediated mRNA degradation is highly conserved in metazoans (3, 4). Additionally, Ccr4-Not regulates RNA polymerase II (Pol II)-dependent transcription initiation and elongation, and mRNA nuclear export (3, 5, 6), while it also controls RNA Polymerase I (Pol I) transcription of ribosomal RNA genes downstream of the target of rapamycin complex 1 (TORC1) signaling pathway (7). Although Ccr4-Not has important transcriptional activities, its mechanistic roles in Pol I and Pol II transcription are less defined compared to its role in mRNA degradation.

While the Ccr4 deadenylase remains the best characterized enzymatic subunit in the complex, Not4 is a highly conserved ubiquitin ligase whose *in vivo* functions are poorly understood. Not4 has an N-terminal RING domain that interacts with the paralogous Ubc4 and Ubc5 E2 enzymes to mediate direct substrate ubiquitination (8). The best characterized Not4 substrates are the histone demethylase Jhd2 and the Cyclin C subunit of the Mediator complex, and Not4-dependent ubiquitination stimulates their degradation by the proteasome (9, 10). Immediately C-terminal to the Not4 RING domain is a highly conserved RNA recognition motif (RRM) and C3H1 motif that are collectively referred to as the RRM-C domain (11), which has homology with other RNA binding proteins. However, while Not4 crosslinks to RNA *in vivo* (12), whether it binds RNA directly through the RRM-C domain is unknown. Not4 RING mutants alone do not completely inactivate Not4 *in vivo*, yet combining both RING and RRM-C mutations simultaneously does phenocopy a Not4 gene deletion mutant (*not4Δ*) (11). Therefore, both domains are required for Not4 *in vivo* function, and the combination of RING and RRM-C domains also makes Not4 a completely unique ubiquitin ligase in the eukaryotic proteome (13). Besides direct substrate ubiquitination, Not4 also behaves as an adaptor *via* recruitment of the HECT-domain ligase Rsp5/NEDD4 (14). This capacity to recruit Rsp5/NEDD4 has the potential to expand Ccr4-Not’s ubiquitin signaling role into areas previously not associated with Ccr4-Not. For example, while Rsp5 regulates transcription and mRNA export (15, 16, 17), it also controls protein sorting and degradation through the endolysosomal compartment and it regulates autophagy (18, 19, 20, 21, 22, 23).

Additional Not4 studies have revealed that it controls translation (24, 25, 26), proteostasis (27, 28, 29), and endolysosomal-mediated nutrient signaling (30), but how Not4 functions in each of these processes remains incompletely understood. Not4 monoubiquitinates both the ribosomal protein Rps7a and the Egd1 and Egd2 (Egd1/2) subunits composing the nascent polypeptide-associated complex (NAC) that mediates co-translational quality control (24, 26). This ubiquitination alters the substrate’s function without signaling for their degradation to regulate translation elongation (31). Loss of the Not subunits, including Not4, reduces polysome formation and alters the ratios of the 40S, 60S, and 80S ribosomes (24), indicating a role in ribosomal stability. Beyond translation, Not4 ligase signaling maintains global proteostasis by ubiquitinating the 19S proteasome subunit Rpt5 (27, 28). Not4 deficient cells assemble proteasomes incorrectly which results in proteasomes with dysregulated deubiquitinase and degradation activity (11, 27, 28, 29). These aberrant proteasomes also become sensitive to vacuole degradation during nutrient stress through a specialized autophagy process termed proteaphagy (29). Recent evidence further connects Ccr4-Not to the autophagy pathway since Ccr4-deficient cells deregulate autophagy gene transcripts and activate macroautophagy in nutrient-replete conditions (32). Ccr4-Not also activates TORC1 signaling by maintaining the vacuole V-ATPase H^+^ pump activity required for TORC1 activation (30, 33, 34, 35). Importantly, Ccr4-Not mutants exhibit synthetic sick or lethal genetic interactions with additional endolysosomal pathway effectors (8, 36, 37, 38), indicating that Ccr4-Not contributes further to endolysosomal regulation through mechanisms not yet understood.

The effect individual Ccr4-Not subunits have on the transcriptome has been described (30, 39, 40, 41), yet the impact that Not4 ligase signaling has on the proteome remains unclear. How the Ccr4-Not ligase recognizes substrates for ubiquitination is unknown, and only a few substrates have been identified. To address this deficiency, we have performed tandem mass tag (TMT) labeling and quantitative mass spectrometry proteome analysis using *not4Δ* reconstituted with a control vector, or vectors overexpressing wild-type (WT) Not4 or an inactive Not4 mutant that phenocopies *not4Δ* (11). The underlying rationale for this approach was to identify proteins whose expression is repressed by Not4 but not by the Not4 mutant, which we have identified and reported in this study. This work has led to the unexpected discovery that both Not4 ligase inactivation and Not4 overexpression inhibit the expression of ribosomal protein (RP) and ribosomal biogenesis (Ribi) factors, which have important functional consequences for protein synthesis and proteostasis. Additionally, we find that the Ccr4-Not ligase actively represses 40S ribosome autophagy, and we provide genetic evidence that suggests both the RP repression and 40S autophagy activation is related functionally to alterations in the endolysosomal pathway.

## Results

### Both increased Not4 expression and loss of its ligase activity inhibit RP and Ribi protein expression

**Figure 1:**
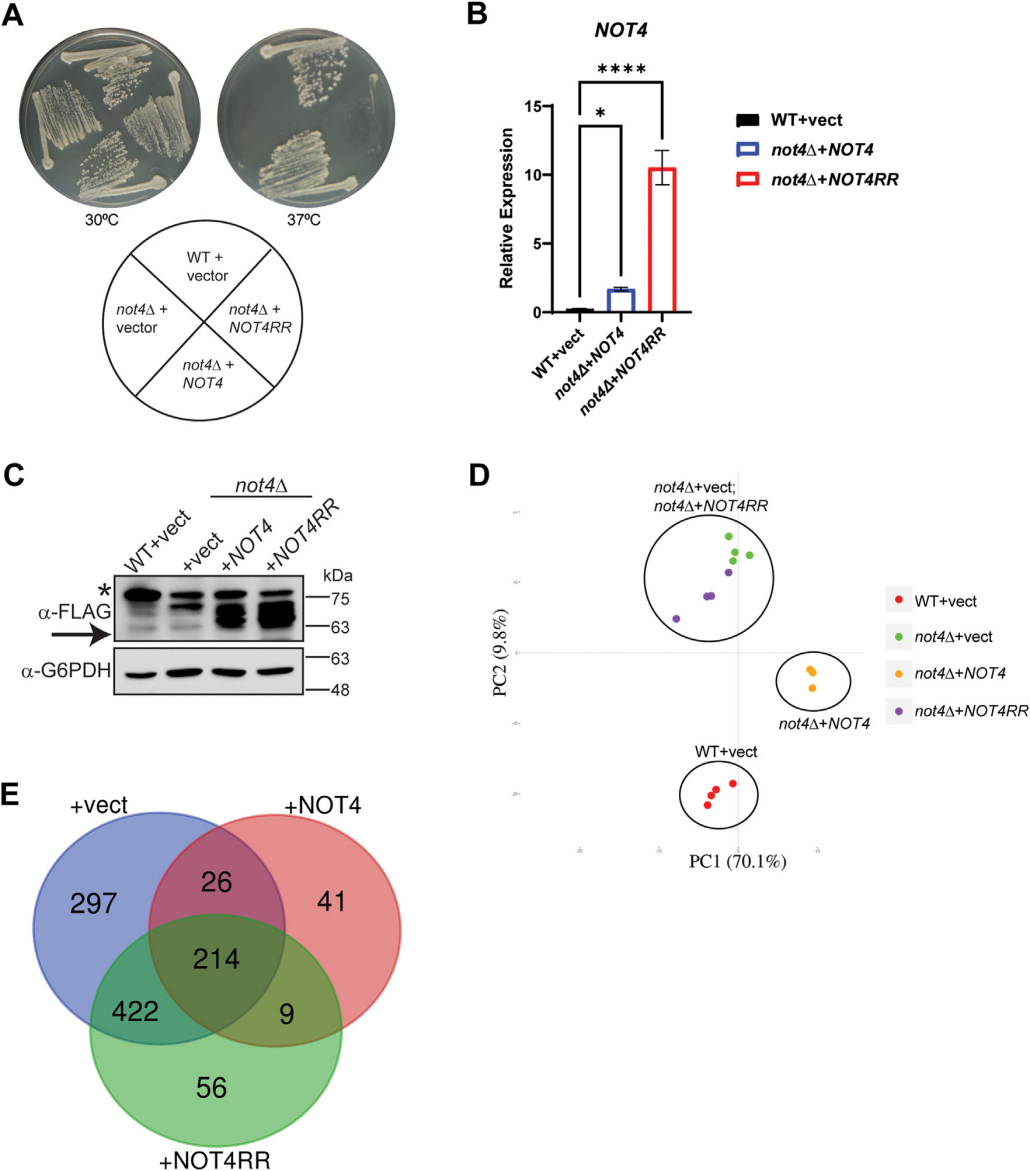
**Not4 ligase levels and activity differentially regulate the cellular proteome.***A*, wild-type (WT) cells transformed with control vector, and *not4Δ* cells expressing either control, *NOT4*, or *NOT4RR* vector, were streaked to duplicate plates and incubated at control temperature (30 °C) or heat shock temperature (37 °C) for 3 days before image capture. *B*, reverse transcriptase coupled with quantitative PCR (RT-qPCR) analysis of *NOT4* transcript levels from the indicated strains. The *NOT4* specific signal was normalized to the expression of the internal control gene *SPT15* and significance was tested by one-way ANOVA. *∗-p< 0.05* or greater. *C*, immunoblot of 30 μg whole cell extract from the cells used in *B*. *Arrow* indicates the Not4 band and the *asterisk* indicates a non-specific band. *D*, proteome principal component analysis (PCA) of the individual replicates for the indicated strains. Each experimental condition had four independent biological replicates except for *not4Δ* + *NOT4*, which had three independent biological replicates. *E*, the differentially expressed proteins (DEPs) were identified by using a 1.5-fold change in expression and an FDR < 0.05. The resulting DEPs then were subjected to Venn analysis. The total number of DEPs in each condition relative to the WT + vector control is 959 (*not4Δ* + vector), 290 (*not4Δ* + *NOT4*), and 701 (*not4Δ* + *NOT4RR*).

Although Ccr4-Not regulation of the transcriptome has been defined in-depth (39, 40), the impact that the Ccr4-Not ligase has on the proteome remains incompletely defined. Furthermore, most Ccr4-Not ligase studies have used cells either lacking Not4 expression entirely (*not4Δ*) (42), express Not4 partial deletion constructs (14), or have reconstituted *not4Δ* with Not4 RING mutants that do not completely phenocopy *not4Δ* (8). Accurate assessment of Not4 function necessitates using a full-length Not4 ligase that incorporates into Ccr4-Not yet a functionally inactive ligase to avoid indirect effects on Ccr4-Not activity independent of its ubiquitin ligase role. Thus, we transformed wild-type (WT) and *not4Δ* with a control vector, vector overexpressing C-terminal FLAG-tagged WT Not4 (*NOT4*), or a Not4 mutant (*NOT4RR*) that contains mutations in both the RING and RRM-C domains. We previously demonstrated that both Not4 and Not4RR incorporate into Ccr4-Not, yet Not4RR is functionally null and phenocopies *not4Δ* (11). Plasmid expressed *NOT4* restored all *not4Δ* phenotypes, including sensitivity to heat stress, while *NOT4RR* failed to restore any *not4Δ* phenotype tested (Fig. 1*A* and see below) (11). Importantly, plasmid expressed *NOT4* and *NOT4RR* transcripts are expressed at higher levels relative to *NOT4* expressed from its endogenous chromosomal locus (Fig. 1*B*), and both Not4 and Not4RR are readily detectable by anti-FLAG immunoblot (IB) (Fig. 1*C*). We reasoned this Not4 and Not4RR overexpression system could be used to identify proteins whose abundance is sensitive to Not4 ligase activity and/or expression levels. These proteins then could be pursued as possible novel Ccr4-Not ligase substrates.

To identify the differentially expressed proteins in these conditions, whole-cell extracts (WCEs) from at least three biological replicates from Figure 1*A* were prepared and subjected to LC-MS-MS analysis using Reporter Ions Quantification approach based on Tandem Mass Tag (TMTpro)-labeling. Quantifiable results for over 3300 proteins in each condition were achieved, and principal component analysis (PCA) revealed high reproducibility between the independent replicates as evidenced by their clustering (Fig. 1*D*). Intriguingly, while *not4Δ* + *NOT4* rescues all *not4Δ* phenotypes (Fig. 1*A*) (11), it clusters distinctly from the WT + vector control, which indicates Not4 overexpression does alter the proteome (Fig. 1*D*). The *not4Δ* + vector and *NOT4RR* samples clustered similarly (Fig. 1*D*), which is predicted since the Not4RR mutant phenocopies *not4Δ* (Fig. 1*A*) (11). To identify the differentially expressed proteins (DEPs) between the experimental conditions, the data were analyzed using a 1.5-fold change in expression with an FDR<0.05 relative to the WT + vector control. Using these criteria, 959 (*not4Δ* + vector), 290 (*not4Δ* + *NOT4*), and 701 (*not4Δ* + *NOT4RR*) DEPs were identified (Fig. 1*E*). Venn analysis of these DEPs identified a substantial overlap of 214 proteins between the vector, *NOT4*, and *NOT4RR* expressing cells, thus indicating a protein subset whose expression is sensitive to both the Not4 expression level and its ligase activity (Fig. 1*E*). Pairwise analyses also reveal exclusive overlap between vector and *NOT4* (26 proteins), *NOT4* and *NOT4RR* (9 proteins), and vector and *NOT4RR* (422 proteins) (Fig. 1*E*). Additionally, the *not4Δ* + vector, *NOT4*, or *NOT4RR* cells exhibit condition-specific DEPs, with 297 DEPs in the *not4Δ* vector samples, while 41 and 56 DEPs were specific for the *NOT4* and *NOT4RR* samples, respectively (Fig. 1*E*). These data indicate that both Not4 expression and its ligase activity have overlapping as well as selective proteome effects. Importantly, we find that the *not4Δ* + vector exhibits reduced expression (greater than 1.5-fold change) of the Caf40 and Not2 Ccr4-Not subunits compared to the WT + vector control while reconstituting cells with either Not4 or the Not4RR mutant restores Caf40 and Not2 protein expression (less than 1.5-fold change for either) (Fig. S1). These data are consistent with the observation that Ccr4-Not subunit loss can destabilize additional complex members (43). While some of the differentially expressed proteins may be due to non-specific effects caused by overexpressing Not4 or the Not4RR mutant, the identified DEPs specifically in Not4 overexpressing cells provide an initial set of factors to test in future efforts to determine if they are candidate Not4 substrates. The protein differences between *not4Δ* + vector and *not4Δ* + *NOT4RR* also validate the rationale for performing the proteome analyses in the presence of a full-length, functionally inactive Not4 ligase mutant to avoid altering the Ccr4-Not complex and affecting additional Ccr4-Not activities that are independent of its ubiquitin ligase activity.

**Figure 2:**
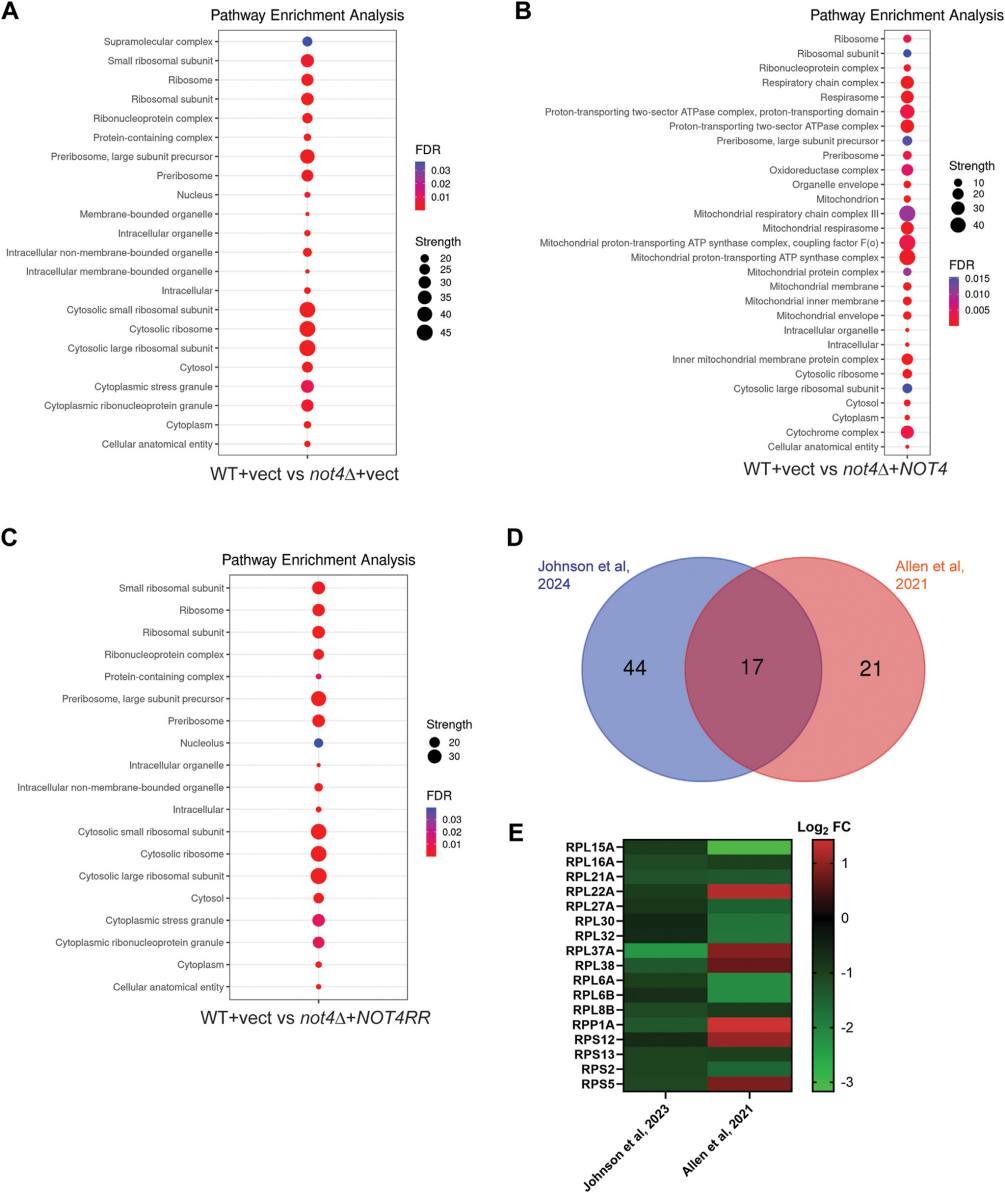
**Ribosomal protein (RP) and ribosomal biogenesis (Ribi) factors are repressed by changes to Not4 activity or expression levels.***A*–*C*, bubble blot analysis of the top cellular component gene ontology (GO) categories for the indicated experimental conditions. GO analysis was performed through the STRING database. File S3 has the GO category and individual protein results used to generate these bubble blots. *D*, Venn analysis of the reduced RPs identified in this study *versus* the RPs differentially expressed in *not4Δ* from the Allen study (42). *E*, heatmap analysis of the Log_2_ expression for each overlapping RP identified in (*D*). File S3 has the RPs listed used for this analysis.

The DEPs for each experimental condition were subjected to Gene Ontology (GO) analysis through the STRING database to identify overrepresented functional networks (44). For *not4Δ* + vector, the GOs were dominated by categories related to the nucleolus, preribosome, and cytoplasmic ribosome (Fig. 2*A*). When these DEPs were further sub-divided into up and down DEPs, the down DEPs were specific for the nucleolar and ribosomal-related GOs, while the up DEP GOs were enriched for proteasome regulatory particle, polarized growth, and cytoskeletal functions (Fig. S2). Ribosomal-specific GOs also were overrepresented in *not4Δ* + *NOT4* expressing cells, although not to the same extent as in *not4Δ* + vector. Additionally, a substantial number of the upregulated *NOT4* GOs related to mitochondrial function and metabolism, while the *NOT4* repressed DEP GOs were specific to ribosomal function (Figs. 2*B* and S2). These data indicate that increased Not4 expression reduces ribosomal factors but enhances the expression of mitochondrial proteins. A similar analysis of the aggregate *not4Δ + NOT4RR* DEPs indicated nucleolar, ribosomal, and cytoplasmic stress granule GOs were overrepresented (Fig. 2*C*). The down DEPs were specific for ribosomal GO categories, while no specific GO categories were overrepresented in the up DEPs (Fig. S2). Collectively, these data indicate that both decreased and increased Not4 activity represses RPs and ribosomal biogenesis (Ribi) factors.

We next examined whether the RP repression was due to inhibition of their mRNA expression. RP genes are coordinately regulated (45), so we chose several RPs from the proteomic dataset and performed RT-qPCR to quantify their mRNA levels. A minor, but statistically significant (*p< 0.05* or greater by two-way ANOVA), decrease in *RPS5* mRNA was found in the *not4Δ* vector, *NOT4*, and *NOT4RR* cells relative to the WT + vector control (Fig. S3). However, the expression of the remaining RP mRNAs either was unaffected or upregulated in these cells relative to the WT + vector control (Fig. S3). As such, these data indicate that RP inhibition in the *not4Δ* + vector, *NOT4*, and *NOT4RR* cells cannot be explained by decreased expression of RP mRNAs. Not4 contributes to translational regulation in part by monoubiquitinating the RP Rps7a and the nascent polypeptide-associated complex factors Egd1 and Egd2 (Egd1/2) to promote co-translational quality control (24, 26). The proteome data revealed that Rps7a and Egd1/2 expression was unaffected in all experimental conditions (less than 1.5-fold change in abundance, see Fig. S4), indicating the global RP inhibition is not caused by repression of known Not4 ribosomal substrates.

The recent study from Allen *et al.* utilizing SILAC analysis reported the proteome differences between WT and *not4Δ* cells and found that the expression of a subset of RPs and translation elongation factors increased in *not4Δ* (42). We compared the RPs identified by the Allen study (proteins represented by a minimum of three peptides and meeting a 1.5 fold-change in expression with a *p*-value *< 0.05*) to the RPs identified in our *not4Δ* + vector dataset. A total of 38 proteins from the Allen study was compared to our results (Table S2), with 21 proteins found to be unique to the Allen study, 44 unique to our dataset, and 17 overlapped (Fig. 2*D*). The overlapping 17 RPs then were compared by plotting their Log_2_ expression to compare the directionality of their expression change. Of these 17 proteins, 11 were decreased in both studies while only six (Rpl22a, Rpl37a, Rpl38, Rpp1a, Rps12, and Rps5) were increased in the Allen study but repressed in our dataset (Fig. 2*E*). Therefore, while our study finds a greater number of RPs to be downregulated in *not4Δ*, for the RPs that do overlap only six are altered in an opposing fashion.

### Loss of Not4 ligase activity impairs protein synthesis and induces 40S RP degradation

**Figure 3:**
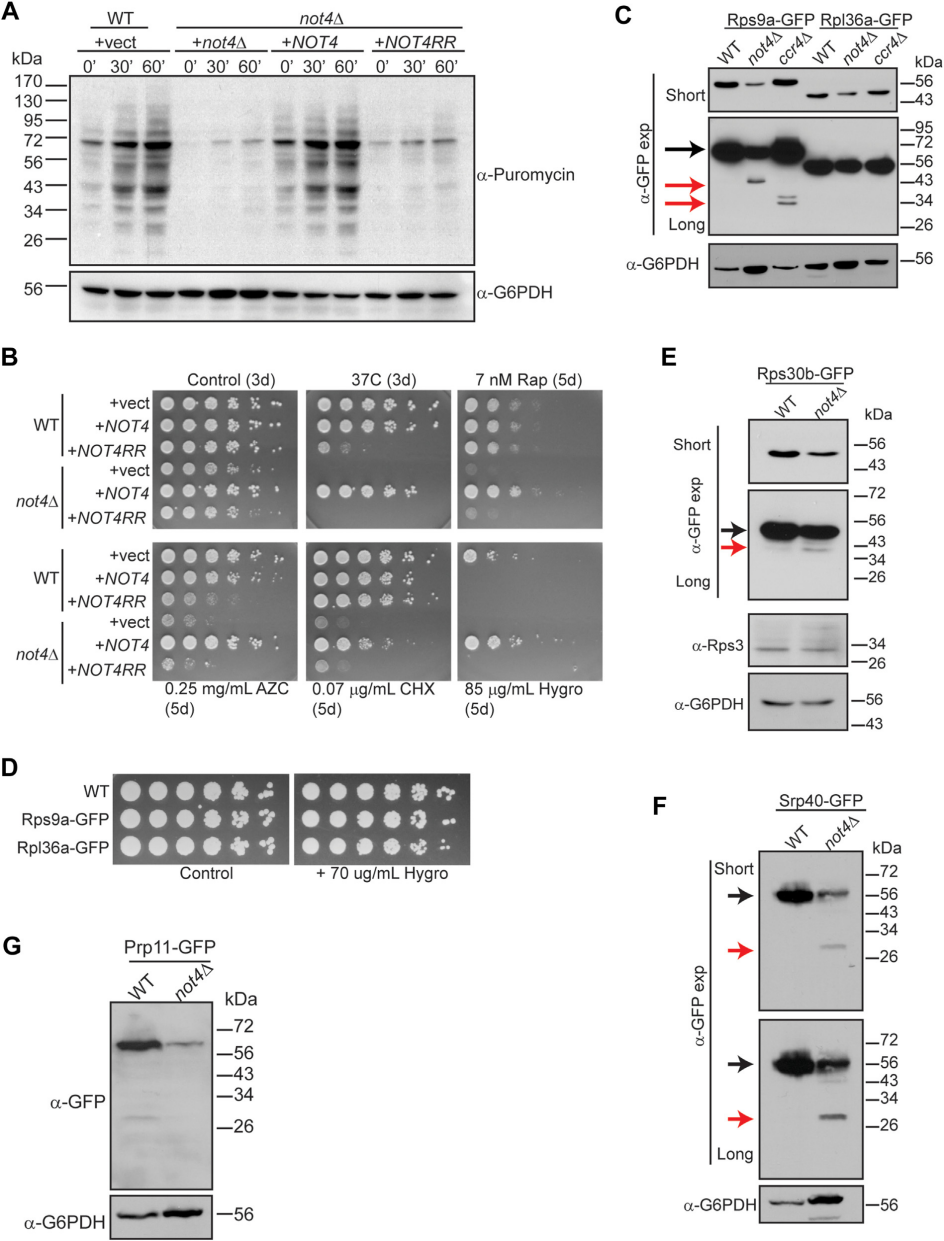
**Both Not4 ligase activity and expression level inhibit RPs and affect ribosomal function *in vivo*.***A*, the indicated cells were grown to log phase and labelled with 10 μM puromycin before taking samples at the indicated timepoints. Whole cell extracts (WCEs, 30 μg) then were analyzed by α-puromycin immunoblot (IB). The α-G6PDH IB serves as a loading control. *B*, WT and *not4Δ* transformed with the indicated expression vectors were cultured overnight at 30 °C. Equal numbers of cells then were 5-fold serially diluted and spotted onto the indicated plates. The number of days the plates were incubated at either 30 °C or 37 °C before pictures were taken are indicated in parentheses. *C*, WCEs (30 μg) from the indicated strains in the Rps9a-GFP or Rpl36-GFP reporter backgrounds were analyzed by α-GFP and α-G6PDH IB. *D*, as in (*B*) except the indicated strains were spotted to control plates or plates containing 70 μg/ml Hygromycin B. *E–G*, As in (*C*) except Rps30b-GFP and Rps3 (*E*), Srp40-GFP (*F*), or Prp11-GFP (*G*) were analyzed.

Ccr4-Not mutants, including *not4Δ*, reduce polysome formation and alter the relative ratios of 40S and 60S ribosomes (24). To test if the RP repression detected was associated with effects on protein synthesis, the WT control, and the *not4Δ* vector, *NOT4*, and *NOT4RR* expressing cells were metabolically labeled with puromycin. Puromycin incorporates nascent protein chains to block protein synthesis, and metabolic labeling of cells with puromycin can be detected by α-puromycin IB, which is used as a readout of active protein synthesis (46). A time-dependent increase in puromycin incorporation was readily detectable in the WT + vector control cells, while puromycin incorporation was reduced dramatically in both the *not4Δ* + vector and *NOT4RR* samples (Fig. 3*A*). Although *NOT4* overexpression inhibits a large network of RPs and Ribi factors (Figs. 2 and S5), *NOT4* restored protein synthesis to the WT state (Fig. 3*A*). Therefore, the ribosomal inhibition in *NOT4* cells is insufficient to disrupt protein synthesis, while protein synthesis in *not4Δ* and *NOT4RR* expressing cells is profoundly inhibited.

The impact Not4 expression and ligase activity have *in vivo* was probed further by analyzing sensitivity to proteostatic stress (incubation at 37 °C and sensitivity to azetidine-2-carboxylic acid (AZC) which causes protein folding stress), nutrient stress (the TORC1 inhibitor rapamycin), or translational stress (cycloheximide and Hygromycin B) (11). For an additional comparison, WT cells also were transformed with the *NOT4* or *NOT4RR* expressing plasmids. As previously reported (11), *not4Δ* vector and *NOT4RR* cells exhibited marked sensitivity to the stress conditions tested, while *NOT4* rescued all of these phenotypes (Fig. 3*B*). In WT cells, *NOT4RR* expression sensitized cells to proteostatic stress (heat stress and AZC), but it had no effect on nutrient stress or cycloheximide-induced translational stress (Fig. 3*B*). These results indicate that Not4RR functions as a dominant negative in WT cells by competing with endogenous Not4 for Ccr4-Not incorporation, suggesting that Ccr4-Not’s role in proteostasis is more sensitive to Not4 ligase activity compared to other stress responses. Intriguingly, while *NOT4* had no effect on the sensitivity of WT cells to most stressors, both *NOT4* and *NOT4RR* profoundly inhibited WT growth in the presence of Hygromycin B (Fig. 3*B*). Hygromycin B stabilizes tRNA interactions with the ribosomal A-site to inhibit tRNA translocation (47), while cycloheximide binds the ribosomal 60S E-site to block ribosome elongation (48). These results suggest that both increased and decreased Not4 ligase activity negatively affects translational control, possibly through mechanisms affecting tRNA-ribosome interactions. Overall, these data suggest that Not4-dependent proteostasis regulation is more dependent on Not4 activity relative to other stress responses and that increased Not4 expression sensitizes cells to mechanistically selective translational inhibitors.

To further delineate Not4-dependent effects on RP expression, we chose two representative RPs, the 40S subunit Rps9a and the 60S subunit Rpl36a, that are contained in the 214 overlapping DEPs for all conditions (Fig. 1*D*). An EGFP epitope tag was integrated in-frame at their genetic loci in WT, *not4Δ*, and *ccr4Δ* such that Rps9a and Rpl36a are expressed as in-frame C-terminal GFP fusions from their native promoters. Using these reporters, we found *not4Δ* reduces the expression of both RPs, as predicted from the proteomic results, while *ccr4Δ* had no effect on either RP (Fig. 3*C*, short α-GFP exposure). These data confirmed that RP inhibition in *not4Δ* is independent of Ccr4-dependent transcription or mRNA deadenylation. While Rps9-GFP is ∼56 kDa, longer exposure of the α-GFP IB revealed a discrete, faster migrating Rps9a-GFP band in *not4Δ* at ∼40 kDa and a doublet at ∼27 to 34 kDa in *ccr4Δ* (Fig. 3*C*). These smaller Rps9a-GFP bands are absent from the WT, and smaller RP-specific bands are not present in the WT, *not4Δ*, or *ccr4Δ* Rpl36a-GFP expressing cells (Fig. 3*C*). These results suggest that the 40S RP may be partially degraded in Ccr4-Not deficient cells. We confirmed that these GFP tags did not increase the cell’s sensitivity to Hygromycin B-induced translational stress (Fig. 3*D*). In addition, analysis of the WT Rps9a-GFP strain by confocal microscopy demonstrates that Rps9a-GFP is broadly found throughout the cell as predicted (Fig. S6), indicating that the GFP tag does not disrupt Rps9a distribution to cause its degradation. Collectively, these data indicate that these Rps9a-GFP and Rpl36a-GFP effects are not due to the GFP tag causing impairment in ribosome function, but instead, they reflect alterations specifically due to loss of the Not4 ligase.

To confirm the inhibitory effects that Not4 loss of function has on additional RPs and Ribi factors identified in our proteomics, we integrated in-frame C-terminal GFP tags at the genes encoding an additional 40S RP gene (*RPS30B*), a Ribi factor (*SRP40*), and a non-ribosomal related spliceosomal factor (*PRP11*) (49). The *not4Δ* repressed full-length Rps30b-GFP expression compared to WT as predicted by our proteomics (Fig. 3*E*). Although not as pronounced as the *not4Δ* Rps9a-GFP degradation product (Fig. 3*C*), we also detected an Rps30b-GFP degradation product that migrates at approximately the same position (∼43 kDa) (red arrow, Fig. 3*E*). To confirm that the *not4Δ* 40S RP repression is specific only for those RPs identified in our dataset, we utilized an antibody recognizing the endogenous 40S subunit Rps3 (not repressed in any of the experimental conditions) and confirmed that *not4Δ* does not affect its expression (Fig. 3*E*). Intriguingly, while *not4Δ* also represses Srp40-GFP as predicted, we also detected a substantial GFP-specific degradation product at ∼27 kDa, which is the size of free GFP (Fig. 3*F*). These partial and/or complete degradation products are specific for the 40S RPs and Ribi factor since *not4Δ* represses Prp11-GFP expression, consistent with our proteomic results, but it does not cause Prp11-GFP degradation (Fig. 3*G*). A recent report demonstrated that *ccr4Δ* upregulates basal macroautophagy in repressive (nutrient-rich) conditions (32). Yeast autophagy flux is assessed by fusing GFP to candidate autophagy substrates and monitoring the release of free GFP (∼27 kDa) that occurs when autophagy cargoes are degraded in the vacuole since GFP resists vacuolar degradation (50). The faster migrating Rps9a-GFP, Rps30b-GFP, and Srp40-GFP bands indicate that under these nutrient-rich repressive conditions, Ccr4-Not ligase loss may increase 40S and Ribi, but not 60S, autophagy (referred to collectively as ribophagy) (51).

**Figure 4:**
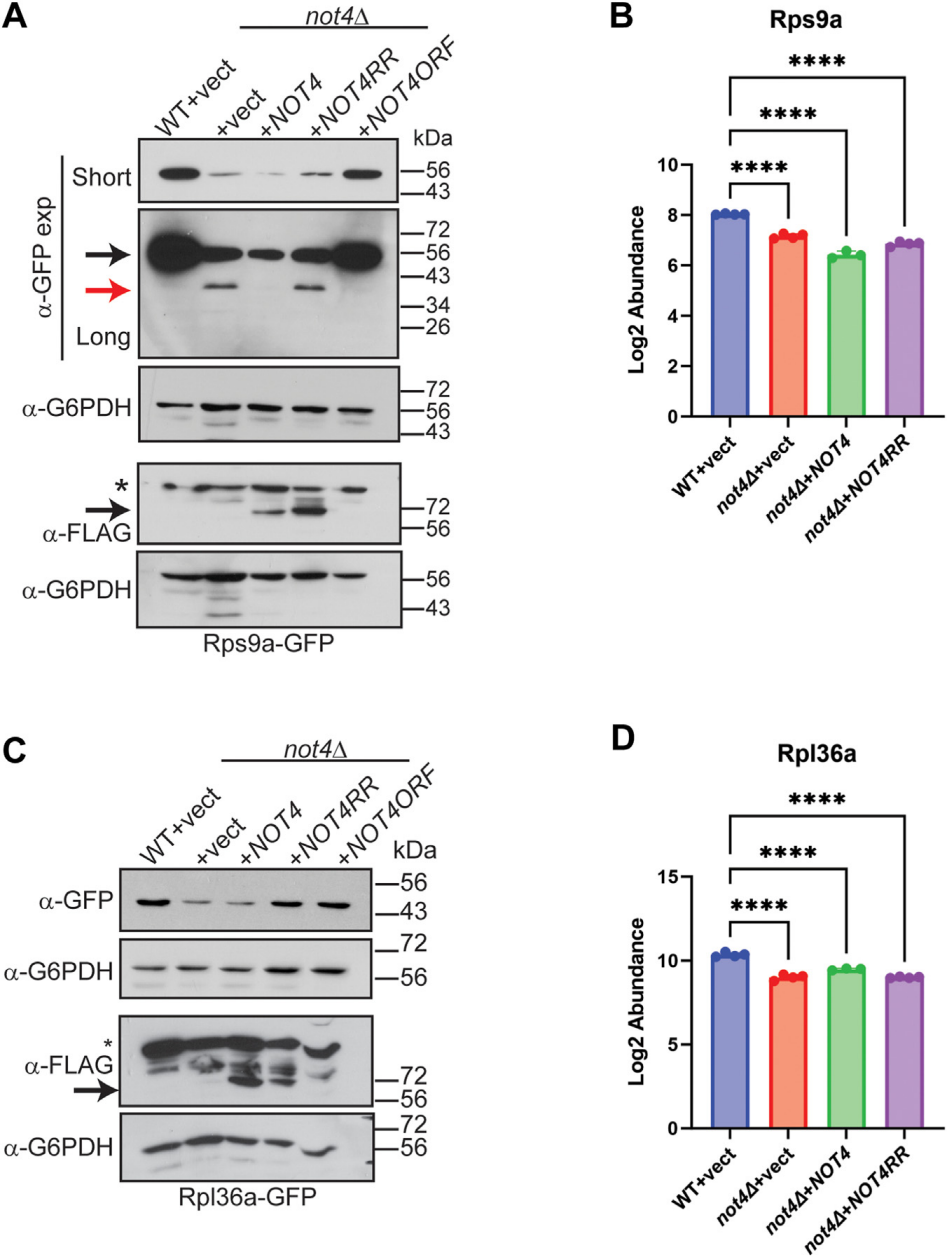
**Not4 expression level and ligase activity differentially affect 40S RP expression and degradation.***A*, duplicate IBs of WCEs (30 μg) from the indicated strains were probed with α-GFP to detect Rps9-GFP and α-FLAG to detect Not4 or Not4RR expression. The α-G6PDH IBs serve as the loading control for each. Short and long exposures for the α-GFP IB are presented. *Black arrow* indicates full-length Rps9a-GFP and *red arrow* indicates partially degraded Rps9-GFP. The ∗ in the α-FLAG IB indicates a non-specific band. *B*, the mean and SD of the peptide abundance signal from the proteomic data for Rps9a of the individual replicates. *∗∗∗∗p < 0.001* by one-way ANOVA. The Log_2_ fold-change (FC) for the individual *not4Δ* samples relative to the WT + vector are as follows: +vector (−0.87); +*NOT4* (−1.6); +*NOT4RR* (−1.2). *C*, same as in (*A*) except Rpl36a-GFP is analyzed. *D*, the mean and SD of the peptide abundance signal from the proteomic data for Rpl36a of the individual replicates. *∗∗∗∗p < 0.001* by one-way ANOVA. The Log_2_ fold-change FC for the individual *not4Δ* samples relative to the WT + vector are as follows: +vector (−1.34); +*NOT4* (−1.8); +*NOT4RR* (−1.34).

The effect that Not4 ligase activity and expression level has on Rps9a-GFP and Rpl36a-GFP also was assessed directly by transforming the WT and *not4Δ* Rps9a-GFP and Rpl36a-GFP reporter strains with control, *NOT4*, or *NOT4RR* expressing plasmids. In addition, a different plasmid was transformed into *not4Δ* that expresses *NOT4* from its endogenous promoter (*NOT4ORF*) so that *NOT4* expression mirrors that of WT cells. Rps9a-GFP remained repressed in the *not4Δ* vector, *NOT4*, and *NOT4RR* cells (Fig. 4*A*), which replicates the results from the proteomic analysis (Fig. 4*B*), while the *NOT4ORF* restored Rps9a-GFP back to WT levels (Fig. 4*A*). Furthermore, the partial Rps9a-GFP degradation product in the *not4Δ* vector and *NOT4RR* samples was absent in both *NOT4* and *NOT4ORF* cells even though *NOT4* overexpression does not restore Rps9a-GFP expression (Fig. 4*A*). In both *not4Δ* vector and *NOT4* overexpressing cells, Rpl36a-GFP was decreased compared to WT (Fig. 4*C*), which is consistent with our proteome results (Fig. 4*D*), while *NOT4ORF* restored Rpl36a-GFP levels back to the WT state (Fig. 4*C*). Interestingly, *NOT4RR* also restored Rpl36a-GFP expression back to WT levels (Fig. 4*C*), which differs from our proteome results (Fig. 4*D*). The underlying explanation for this discrepancy currently is unclear. This effect likely stems from an unknown effect the GFP tag has on Rpl36a regulation even though the Rpl36a-GFP tag does not alter sensitivity to ribosome stress (Fig. 3*D*). Collectively, these data indicate that a narrow range of Not4 ligase activity maintains wild-type RP expression, and these RP effects encompass both 40S and 60S RPs. They also indicate that the partial Rps9a-GFP degradation in *not4Δ* specifically results from a lack of Not4 ligase activity independently of Not4-mediated Rps9a repression.

### Not4 ligase loss represses 40S RP expression independent of proteasome-mediated degradation but dependent on the endolysosomal pathway

**Figure 5:**
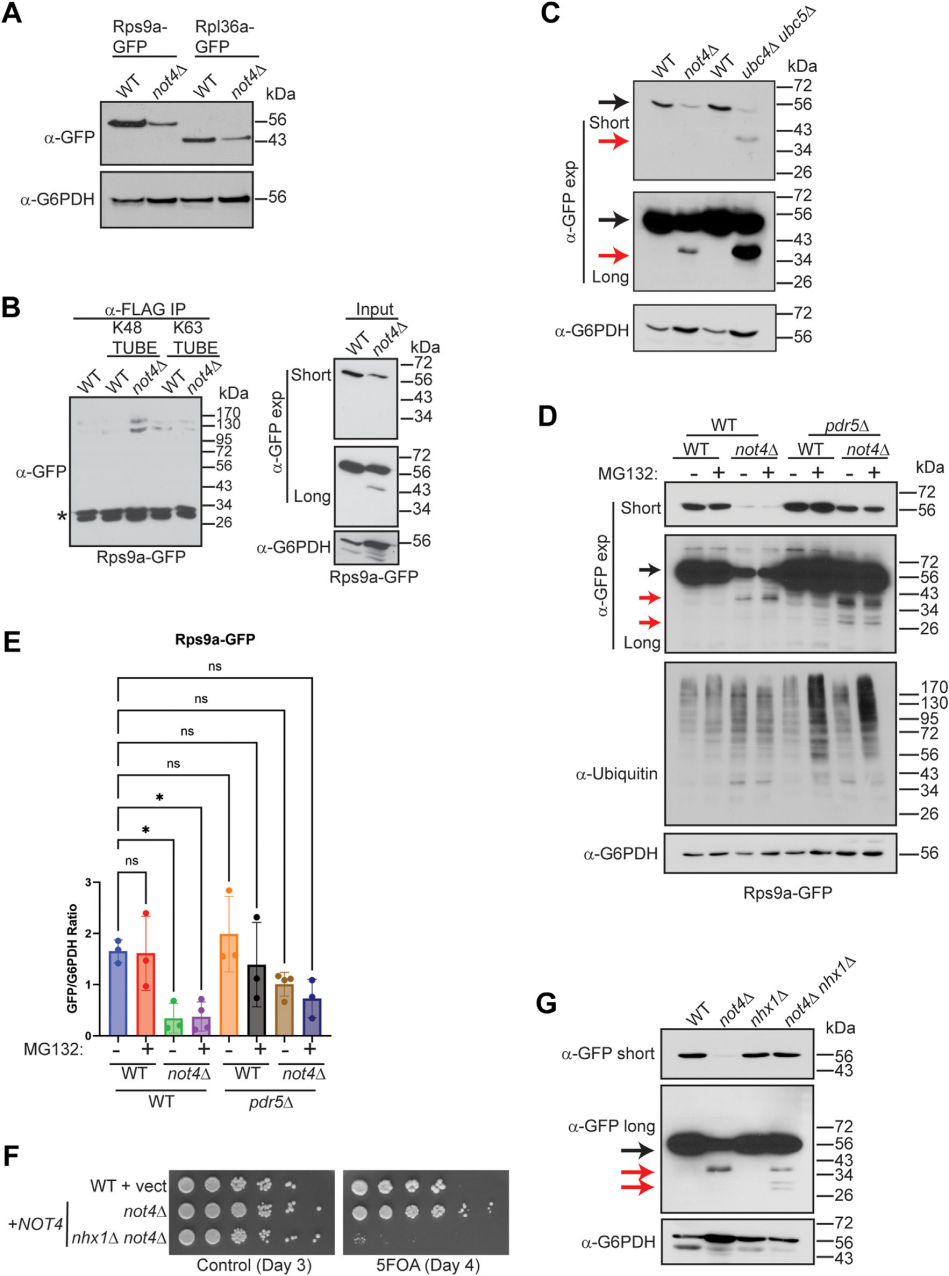
**Not4 ligase loss causes Rps9a degradation independently of the proteasome but dependent on the endolysosomal pathway.***A*, equal numbers of WT and *not4Δ* were lysed by boiling in SDS-PAGE loading buffer and then analyzed by α-GFP IB. The α-G6PDH IB is the loading control. *B*, all WCEs (600 μg) from the indicated strains were treated with α-FLAG antibody combined in the absence or presence of 3 μg of either K48 or K63-specific TUBES. After incubation with rotation at 4 °C for 2 h, protein A conjugated agarose beads were added and incubated for an additional 1 h. The bead-immune complexes then were pelleted and washed extensively before analyzing by 10% SDS-PAGE and α-GFP IB. The inputs represent 30 μg of the WCEs. Short and long α-GFP exposures are provided. The α-G6PDH IB serves as the loading control. ∗- non-specific band. *C*, log phase WCEs (30 μg) were prepared from the WT and indicated single or double mutants and analyzed by α-GFP. *D*, the indicated strains were grown to log phase and then mock treated or treated with 10 μM MG132 for 2 h to inhibit the proteasome. Short and long α-GFP IBs are provided. *Black arrow* indicates full-length Rps9a-GFP and *red arrows* indicate partial and complete degradation products. The α-G6PDH IB is the loading control. *E*, quantitation of (*D*). The short α-GFP IBs for a total of 3 to 4 individual biological replicates were quantified and expressed as the ratio of full-length GFP to G6PDH. *∗p < 0.05* by one-way ANOVA. *F*, the WT and *not4Δ* strains were transformed with control or *NOT4* expression vector. Equal numbers of cells from overnight cultures then were 5-fold serially diluted and spotted to either plasmid-selective media or media containing 5-FOA and images were captured on the indicated days. *G*, log phase WCEs (30 μg) from the indicated WT, single, and double mutants were IB with α-GFP. Short and long exposures are provided with the *black arrow* denoting full-length Rps9a-GFP and the *red arrows* indicating degradation products. The α-G6PDH IB is the loading control.

Recent studies determined that loss of Not4-mediated ribosomal Rps7a monoubiquitination causes defects in protein solubility and increases protein aggregate formation (24, 25). To test for this possibility, equal numbers of WT and *not4Δ* cells expressing Rps9a-GFP or Rpl36a-GFP were lysed by boiling in SDS-PAGE sample buffer and then analyzed by α-GFP IB. Even when samples were prepared under denaturing conditions, *not4Δ* repressed both RPs (Fig. 5*A*). Importantly, overexpressing WT Not4 still inhibits a large network of RP and Ribi factors (Figs. 2 and S5) although it fully restores the protein synthesis defects (Fig. 3*A*), so the RP inhibition detected cannot be explained by the aggregate formation and protein insolubility.

RPs produced in excess beyond that needed for ribosome biogenesis are flagged with ubiquitin and degraded through the ubiquitin-proteasome (UPS) system (52, 53). Using Rps9a as a model RP, we assessed if Rps9a was ubiquitinated in *not4Δ* cells to promote RP repression through proteasome-mediated degradation. Initially, WCEs from WT and *not4Δ* were treated with FLAG-tagged K48 or K63 linkage-specific Tandem Ubiquitin Binding Entities (TUBEs) before performing α-FLAG pull-down assays and Rps9a detection by α-GFP IB (54). While only background levels of a high molecular weight (∼130–170 kDa) Rps9a-GFP specific signal was detected in the control and WT K48-TUBE specific IPs, the Rps9a-GFP signal increased specifically in the *not4Δ* K48-TUBE IP (Fig. 5*B*). Separately, the K63-TUBE IPs pulled down K63-linked ubiquitinated Rps9a above the background from WT, yet *not4Δ* did not alter its abundance (Fig. 5*B*). These data suggest that Not4 loss selectively increases K48-linked Rps9a ubiquitination. Importantly, the high molecular weight Rps9a-GFP signal is specific since the Rps9a-GFP signal was undetectable in the normal Rps9a-GFP size range of ∼56 kDa, thus indicating Rps9a is modified by multiple ubiquitin chains (Fig. 5*B*). While *not4Δ* results in incomplete Rps9a-GFP degradation, *ccr4Δ* results in mostly complete degradation and liberation of free GFP (Fig. 3*C*). How Not4 recognizes substrates is unknown, but a previous study determined that Ccr4 contributes to Not4-mediated ubiquitin signaling (55), suggesting that *ccr4Δ* likely impairs Not4-dependent inhibition of Rps9a-GFP degradation. To explore this possibility further, we tested if the Rps9a-GFP inhibition and its increased degradation also occurred in cells lacking the paralogous E2 enzymes Ubc4 and Ubc5 utilized by Not4 to mediate substrate ubiquitination (8). Rps9a-GFP expression was reduced similarly in both *not4Δ* and *ubc4Δ ubc5Δ* compared to WT cells, while *ubc4Δ ubc5Δ* caused even greater Rps9a-GFP partial degradation compared to *not4Δ* (Fig. 5*C*). Since Ubc4 and Ubc5 are not Ccr4-Not subunits, these data further support the concept that *ccr4Δ* impairs a Not4 ligase-regulated process that promotes 40S RP expression and inhibits 40S RP degradation. Since several different E3 ligases utilize these E2 enzymes (56), these results also indicate that additional E3 ligases must function redundantly with Not4 in this regard.

Because *not4Δ* increased Rps9a-GFP K48-linked ubiquitination, we tested if *not4Δ* inhibited Rps9a-GFP expression by increasing its proteasome-dependent degradation. WT and *not4Δ* were treated with 10 μM MG132 to inhibit the proteasome. Since WT cells are resistant to proteasome inhibitors, we also engineered a *pdr5Δ* into both WT and *not4Δ* and repeated these experiments. Pdr5 is a plasma membrane efflux pump that exports xenobiotics and H^+^ from cells (57), and its loss sensitizes cells to proteosome inhibitors (58). While MG132 treatment did not inhibit proteasome activity in WT or *not4Δ*, *pdr5Δ* loss sensitized both WT and *not4Δ* to proteasome inhibition indicated by the MG132-dependent increase in global protein polyubiquitination (Fig. 5*D*). Unexpectedly, we found that *not4Δ pdr5Δ* restored Rps9a expression back to near WT levels irrespective of proteasome inhibition (Fig. 5, *D* and *E*). Longer α-GFP exposure also revealed that *not4Δ pdr5Δ* enhanced Rps9a-GFP degradation relative to *not4Δ* such that detectable free GFP (∼27 kDa) accumulated, which was not increased further by proteasome inhibition (Fig. 5*D*). These data reveal that *not4Δ* does not repress this 40S RP or promote its incomplete degradation through a proteasome-dependent mechanism. They also indicate that the combined *pdr5Δ not4Δ* opposes Rps9a repression due to *not4Δ* while it increases Rps9a-GFP degradation to free GFP.

Previously, we found that Ccr4-Not disruption inhibits TORC1 signaling by impairing vacuole V-ATPase activity and reducing vacuole acidity (30). V-ATPase-mediated vacuole acidification is essential for autophagy substrate degradation, nutrient storage and recycling, and the downstream activation of the RP and Ribi gene transcription and mRNA translation necessary for ribosomal biogenesis (59, 60). Since Pdr5 exports H^+^ from cells, we speculated that Pdr5 may oppose vacuole V-ATPase function indirectly by competing for a limiting pool of free cytoplasmic H^+^ (61). If so, then in Ccr4-Not deficient cells where V-ATPase function is compromised (30), Pdr5 loss may alleviate the inhibitory effect that Ccr4-Not disruption has on the V-ATPase by reducing competition for H^+^. To test this possibility, *not4Δ* was combined with *nhx1Δ* that lacks the vacuole Na^+^-K^+^/H^+^ exchanger, and causes vacuole hyperacidification (62). While *not4Δ nhx1Δ* reconstituted with *NOT4* from a *URA3* expression plasmid had no overt growth defects, selection on 5-FOA media, which forces *NOT4* plasmid loss, revealed *not4Δ nhx1Δ* to be synthetically sick compared to *not4Δ* (Fig. 5*F*). However, the *not4Δ nhx1Δ* fully restored Rps9a-GFP expression, and it increased the conversion of the partial Rps9-GFP degradation product (∼40 kDa) to free GFP (∼27 kDa) (Fig. 5*G*). These results indicate that Ccr4-Not disruption represses RP expression and inhibits 40S RP degradation by disrupting V-ATPase-mediated acidification of the endolysosomal compartment. Furthermore, *not4Δ* ribosomal repression cannot be explained as an indirect consequence of poor growth since the *not4Δ nhx1Δ* restores Rps9a expression, yet it is more profoundly growth impaired (Fig. 5*F*).

### 40S ribophagy activation in Ccr4-Not ligase-deficient cells is independent of TORC1 inhibition and the Atg7-dependent macroautophagy pathway

**Figure 6:**
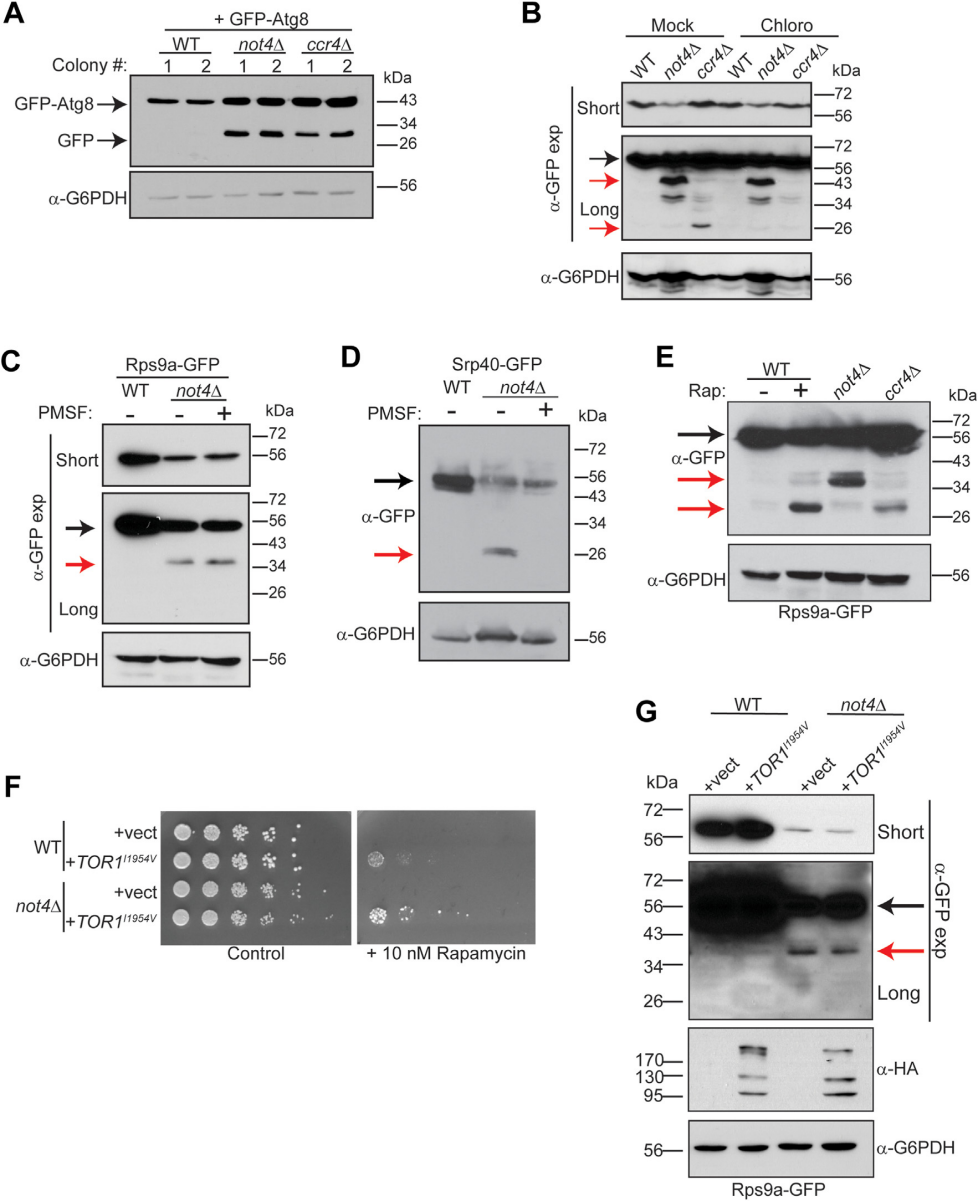
**Ccr4-Not ligase deficiency causes 40S ribophagy independent of TORC1 inhibition.***A*, a plasmid expressing GFP-Atg8 from its native promoter was transformed into WT, *not4Δ*, and *ccr4Δ*. Log phase WCEs from two independent transformants for each strain background were analyzed by α-GFP IB. The GFP-Atg8 and GFP only bands are indicated. *B*, analysis of Rps9a-GFP in WT, *not4Δ*, and *ccr4Δ* mock treated or treated with 5 mM chloroquine (Chloro) for 4 h. WCE were prepared and 30 μg then analyzed by α-GFP IB. The α-G6PDH IB is the loading control. *C*, WT and *not4Δ* Rps9a-GFP cells were mock treated or treated (*not4Δ*) with 1 mM phenylmethanesulfonyl fluoride (PMSF) for 4 h before α-GFP IB as described in (*A*). *D*, as in (*C*) except Srp40-GFP was analyzed. *E*, the WT, *not4Δ*, and *ccr4Δ* strains expressing Rps9a-GFP were grown to log phase before preparing WCEs. In parallel, a log phase WT culture was treated with 200 nM rapamycin (Rap) for hours before harvesting and WCE preparation. Samples (30 μg) were analyzed by α-GFP IB and then α-G6PDH as a loading control. The *black arrow* indicates full-length Rps9a-GFP and the red arrows indicate partial or complete (∼27 kDa) degradation products. *F*, WT and *not4Δ* were transformed with control vector or vector expressing the Tor1^I1954V^ mutant. Equal numbers of cells from overnight cultures were 5-fold serially diluted and spotted to control selection plates or selection plates containing the TORC1 inhibitor rapamycin (10 nM). Images were acquired after 4 days of growth at 30 °C. *G*, the strains from (*F*) were grown to log phase and WCEs (30 μg) then analyzed by α-GFP IB to detect Rps9a-GFP and α-HA to detect Tor1^I1954V^ expression. The α-G6PDH IB is the loading control. Short and long α-GFP IBs are indicated with the *black arrow* denoting full-length Rps9a-GFP and the *red arrow* indicating the partial degradation product.

Our results indicate that the increased K48-linked RP ubiquitination in *not4Δ* does not result in RP proteasomal degradation. Combined with the findings of increased partial (in *not4Δ*) and complete (in *ccr4Δ*) Rps9a-GFP degradation (Fig. 3*C*), we considered whether Ccr4-Not disruption represses RP levels through a macroautophagy-dependent mechanism. Initially, we tested if macroautophagy deregulation occurs in *not4Δ* by transforming WT, *ccr4Δ*, and *not4Δ* with a GFP-Atg8 expressing plasmid (32). GFP is cleaved from Atg8 when autophagosomes fuse with the vacuole, yet GFP resists vacuole degradation, so free GFP accumulation indicates increased macroautophagy (50). Log phase cultures of two independent plasmid transformants from each background were analyzed by α-GFP IB and, consistent with macroautophagy inhibition in nutrient-rich environments, WT cells have no detectable free GFP (Fig. 6*A*). As previously reported (32), *ccr4Δ* increased macroautophagy in nutrient-rich conditions, which is indicated by the presence of free GFP (32), while *not4Δ* also activated macroautophagy (Fig. 6*A*). The *ccr4Δ* deregulates autophagy by stabilizing autophagy (*ATG*) transcripts, so *ATG* transcript levels were measured to determine if Not4 ligase signaling affects their expression similarly. *ATG* transcript levels were unaffected or elevated in both *not4Δ* + vector and *not4Δ* + *NOT4RR* cells, while their expression in *not4Δ* + *NOT4* either was comparable to the WT + control or only slightly upregulated (Fig. S7). Thus, both Ccr4-Not ubiquitin ligase and deadenylase activities repress *ATG* transcript levels in macroautophagy-inhibitory environments. However, it remains unknown if Ccr4-Not ligase signaling accomplishes this by promoting their mRNA decay or through transcriptional repression.

The deregulated macroautophagy in *not4Δ* and *ccr4Δ* suggested that the Rps9a-GFP degradation may be mediated by increased 40S ribophagy. To test this possibility, *not4Δ* and *ccr4Δ* were treated with the lysosomal inhibitor chloroquine which increases vacuole pH and inhibits autophagy (63, 64). Chloroquine treatment reduced Rps9a-GFP degradation and prevented the liberation of free GFP in *ccr4Δ*, indicating that Ccr4-Not disruption mediates 40S degradation *via* vacuole-dependent autophagy (Fig. 6*B*). Intriguingly, chloroquine treatment did not alter the partial Rps9a-GFP degradation in *not4Δ* (Fig. 6*B*). To further probe a role for increased Rps9a-GFP autophagy in *not4Δ*, we treated both WT and *not4Δ* Rps9a-GFP and Srp40-GFP with the distinct autophagy inhibitor (PMSF) as previously reported (65). Consistent with the chloroquine results, PMSF treatment also failed to prevent the *not4Δ* dependent Rps9a-GFP partial degradation, while it completely prevented Srp40-GFP degradation to free GFP (Fig. 6, *C* and *D*). Ccr4-Not disruption decreases V-ATPase-mediated vacuole acidification (30), which is predicted to reduce autophagy efficiency due to the increased vacuole pH. We have observed that *not4Δ* causes greater V-ATPase inhibition relative to *ccr4Δ* (unpublished observations). Therefore, the above results would be consistent with *not4Δ* causing reduced autophagy efficiency of 40S RPs, most likely because they exist complexed with additional RPs, while single Ribi factors are degraded more efficiently.

To further explore the relationship between Ccr4-Not and 40S ribophagy, Rps9a-GFP WT, *not4Δ*, and *ccr4Δ* nutrient-rich cultures were harvested, while a duplicate WT culture was treated with the TORC1 inhibitor rapamycin for 4 h to induce robust ribophagy. Although no Rps9a-GFP ribophagy occurred in the WT control, substantial free GFP (∼27 kDa) accumulated in both the rapamycin-treated WT and *ccr4Δ* cells (Fig. 6*E*). Importantly, an intermediate Rps9a-GFP degradation product was visible in the WT rapamycin-treated sample, matching the size of the partial degradation product in *not4Δ* (Fig. 6*E*). These data provide further support for increased 40S ribophagy-mediated degradation of Rps9a-GFP in Ccr4-Not deficient cells. Since Ccr4-Not disruption inhibits TORC1 signaling (30), we tested if the 40S ribophagy was due solely to impaired TORC1 activity. WT and *not4Δ* were transformed with a control plasmid or plasmid expressing an HA-tagged Tor1^I1954V^ mutant that has increased kinase activity (66). Tor1^I1954V^ rescued WT and *not4Δ* growth in the presence of rapamycin (Fig. 6*F*), thus confirming it enhances TORC1 signaling in both. However, Tor1^I1954V^ failed to restore Rps9a-GFP expression or prevent 40S ribophagy in *not4Δ* (Fig. 6*G*). Therefore, while Ccr4-Not inhibits 40S ribophagy in nutrient-rich environments through a ubiquitin ligase-dependent mechanism, this 40S ribophagy inhibition is not due solely to Ccr4-Not mediated TORC1 activation.

**Figure 7:**
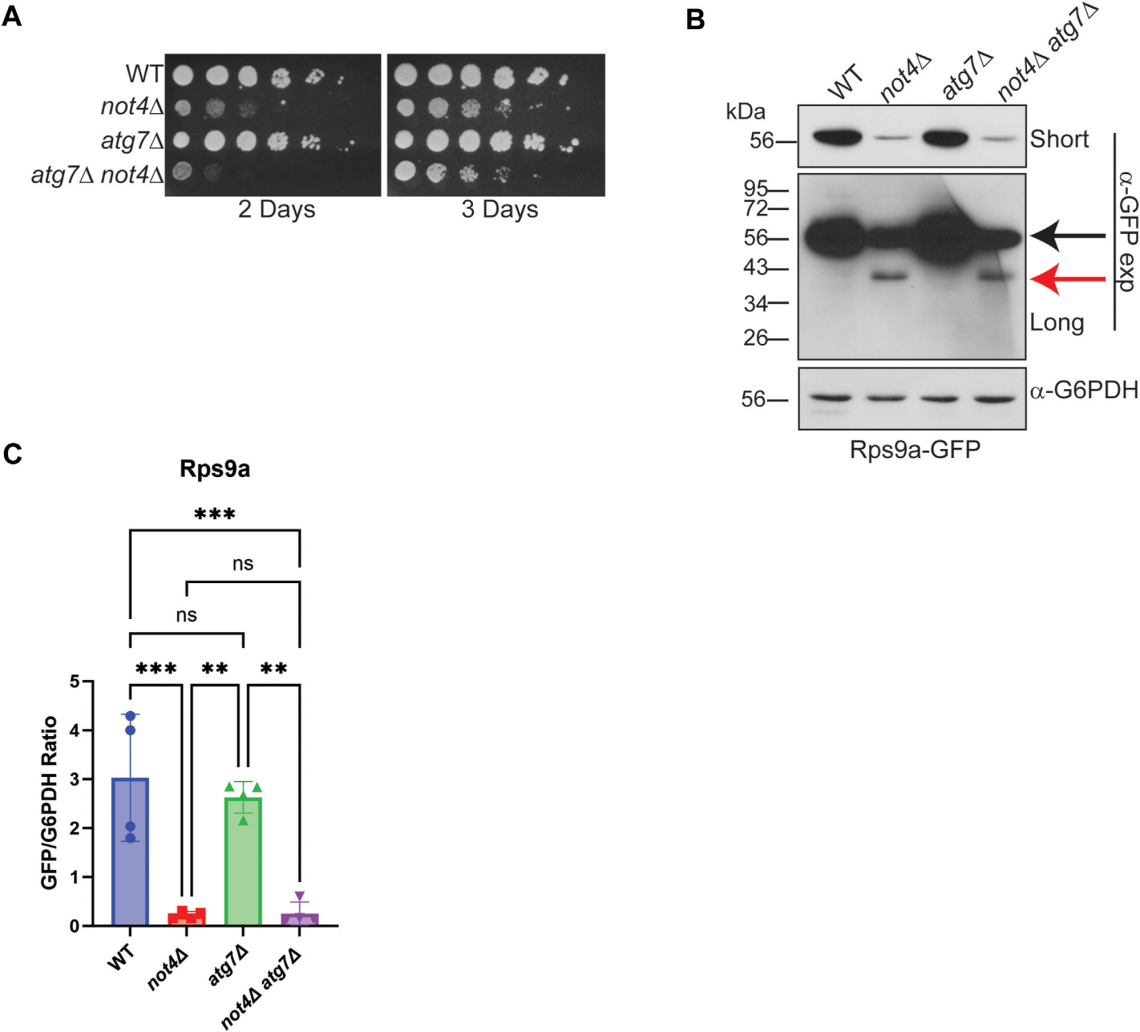
**40S ribophagy activation in Ccr4-Not ligase deficient cells is independent of macroautophagy.***A*, the individual and double mutants were cultured overnight, and then equal cell numbers were 5-fold serially diluted, spotted to YPD plates, and incubated at 30 °C. Images were taken at the indicated times. *B*, Log phase WCEs (30 μg) from the strains in (*A*) were analyzed by α-GFP IB. Short and long exposures are provided with the full-length Rps9a-GFP indicated by the *black arrow* and the degradation product denoted by the *red arrow*. *C*, quantification of (*B*). The short α-GFP IBs for a total of 3 to 4 individual biological replicates were quantified and expressed as the ratio of full-length GFP to G6PDH. *∗∗p< 0.005* or greater by one-way ANOVA.

Previously, *not4Δ* was reported to have a minor synthetic sick phenotype when combined with an *atg7Δ* mutant that blocks autophagosome-dependent macroautophagy (24). While an increase in 40S ribophagy was not reported in this study, a different 40S reporter strain (Rps3-GFP) was used in this analysis. We tested the possibility that the 40S ribophagy in *not4Δ* could be mediated by increased Atg7-dependent macroautophagy by combining *not4Δ* with *atg7Δ*. As previously reported, the *atg7Δ not4Δ* exhibited only a mild synthetic sick phenotype compared to *not4Δ* (Day 2, Fig. 7*A*). However, the *not4Δ atg7Δ* neither restored full-length Rps9a-GFP expression nor prevented Rps9a-GFP partial degradation (Fig. 7, *B* and *C*). Thus, while Ccr4-Not ligase disruption deregulates macroautophagy in nutrient-rich environments (Figs. 3*C*, 4*A*, and 6), both the 40S RP repression and increased 40S ribophagy in *not4Δ* are Atg7 macroautophagy-independent.

### TORC1-stress-induced 40S ribophagy requires a functional ESCRT pathway but redundant pathways mediate 40S ribophagy in a Ccr4-Not ligase mutant

**Figure 8:**
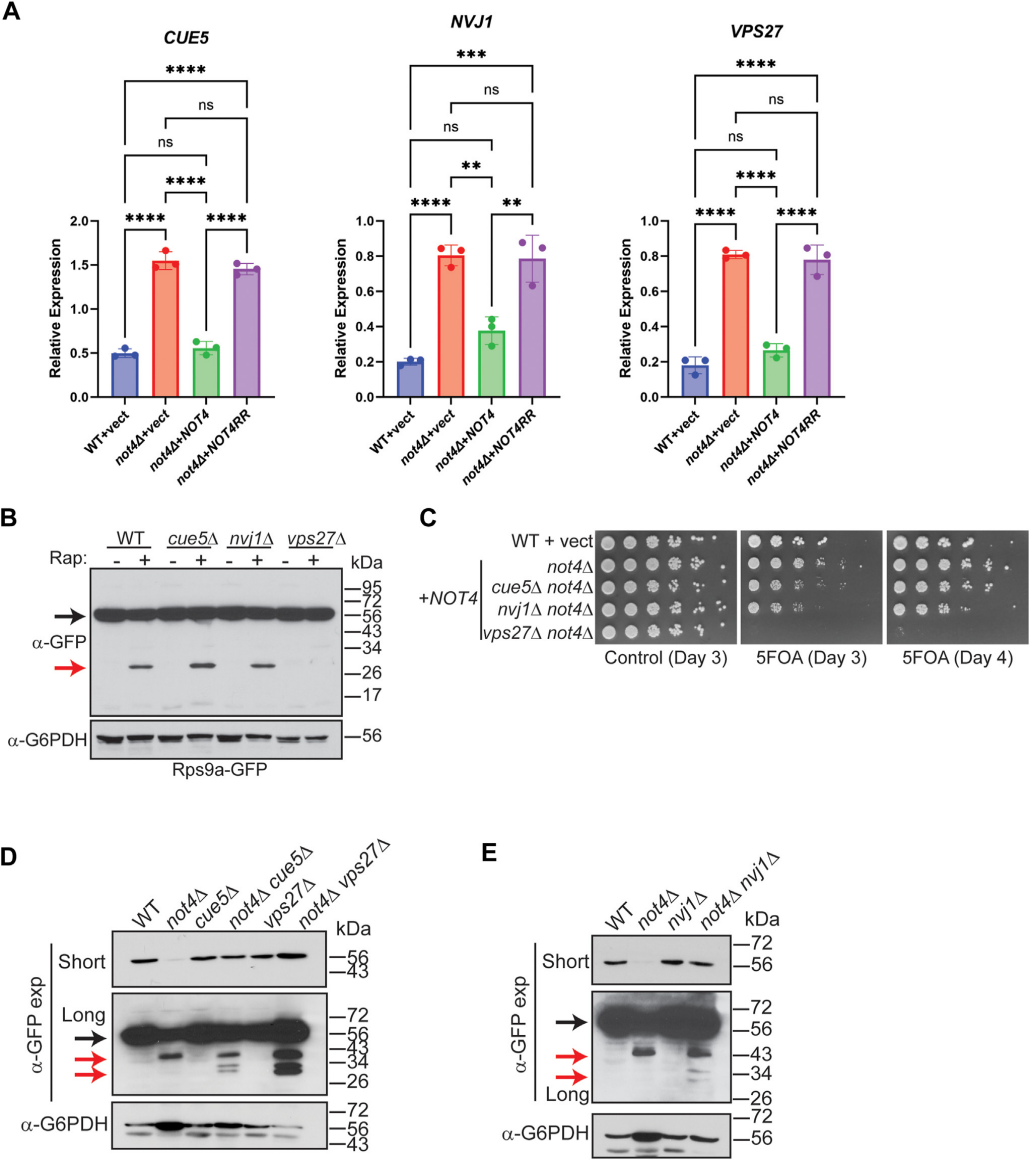
**Both 40S RP repression and ribophagy activation in Ccr4-Not ligase deficient cells are functionally connected to the endolysosomal pathway.***A*, RT-qPCR analysis of the indicated genes from triplicate independent biological replicates with the mean and SD plotted and significance determined by one-way ANOVA. *∗∗p < 0.005* or greater. *B*, the WT and individual mutants were cultured to log phase and mock-treated or treated with 200 nM rapamycin for 4 h before harvesting and performing α-GFP IB. The *black arrow* indicates full-length Rps9a-GFP and the *red arrow* indicates free GFP. *C*, WT cells and the indicated double mutants were transformed with either control or *NOT4* expression vector and were cultured overnight. Equal cell numbers then were 5-fold serially diluted, spotted to plasmid-selective control media or to media containing 5-FOA, and then incubated at 30 °C for the indicated times before photographing. *D* and *E*, the WT, single, and double mutants were grown to log phase and then analyzed by α-GFP IB. Short and long α-GFP exposures are provided. The *black arrow* indicates full-length Rps9a-GFP and the *red arrows* indicate partial and complete GFP degradation products.

The effectors selectively regulating 40S ribophagy remain unknown (51, 67). Because the *not4Δ* vector and *NOT4RR* cells both induced partial 40S ribophagy, we analyzed their overlapping DEPs (Fig. 1*D*) for factors connected either to autophagy or ribosome turnover pathways that may mediate the 40S ribophagy. Three upregulated proteins were identified (Cue5, Nvj1, and Vps27) that have been linked to autophagy previously (Fig. S8). Cue5 is a CUET protein family member that binds ubiquitin and interacts with Atg8 to mediate proteaphagy and protein aggregate autophagy (68, 69, 70). Nvj1 is a nuclear membrane protein that can interact with the vacuole protein Vac8 to promote piecemeal microautophagy of the nucleus (PMN) during nutrient stress (71). Vps27 is a subunit of the endosomal sorting complex required for transport (ESCRT) complex that binds ubiquitinated substrates to promote their degradation *via* autophagosome-independent microautophagy (72), while it also regulates endosomal protein sorting and turnover of plasma membrane proteins (73). Analysis by RT-qPCR revealed that loss of Not4 ligase activity increases the mRNA levels for all three proteins (Fig. 8*A*), indicating that Not4 ligase activity either promotes their mRNA decay or represses their transcription. To determine if they contribute to 40S ribophagy, WT, *cue5Δ*, *nvj1Δ*, and *vps27Δ* were treated with rapamycin for 4 h to induce ribophagy before analyzing Rps9a-GFP cleavage. While no Rps9a-GFP cleavage occurred in mock-treated cells, TORC1 inhibition induced robust Rps9a-GFP cleavage in WT, *cue5Δ*, and *nvj1Δ*, while this cleavage was abolished by *vps27Δ* (Fig. 8*B*). Thus, TORC1 inhibition activates 40S ribophagy dependent on a functional Vps27-dependent ESCRT pathway. 60S ribosomes also undergo nutrient-stress induced ribophagy regulated by the Ltn1 ubiquitin ligase that ubiquitinates the 60S to inhibit 60S ribophagy, while the Ubp3 and Bre5 (Ubp3/Bre5) deubiquitinase removes 60S ubiquitination to activate 60S ribophagy (67, 74). Because 60S ribophagy was not detected in *not4Δ* or *ccr4Δ* (Fig. 3*C*), we inspected the Not4 proteome data to determine if the expression of these 60S ribophagy effectors was altered. Intriguingly, while Ltn1 and Ubp3 protein levels were unaffected, we found that Bre5 expression was repressed significantly, and it is among the 422 proteins that overlap in the *not4Δ* vector and *NOT4RR* cells (Figs. S9 and 1*E*). Overall, these data demonstrate that the ubiquitin-binding ESCRT subunit Vps27 is required for 40S ribophagy activation during nutrient stress, suggesting that the Vps27 upregulation in Ccr4-Not ligase mutants may contribute to 40S ribophagy. Our proteomic results also indicate that 60S ribophagy likely is not induced in Not4-deficient cells due to Bre5 repression.

If Not4-deficient cells increase Vps27 expression as the sole mechanism for inducing 40S ribophagy, then combining *vps27Δ* with *not4Δ* should prevent its occurrence. To test this possibility, *not4Δ* was combined separately into *vps27Δ*, and also into *cue5Δ* or *nvj1Δ* for comparison, and then the growth phenotypes of the single and double mutants were compared. The *cue5Δ not4Δ* and *nvj1Δ not4Δ* all exhibited synthetic sick phenotypes when forced to lose the *NOT4* expressing plasmid on 5-FOA plates, while the *vps27Δ not4Δ* was even more growth impaired (Fig. 8*C*). This extreme synthetic sick phenotype is consistent with a previous report that found mating *vps27Δ* to *not4Δ* failed to generate viable *vps27Δ not4Δ* spores (8). Surprisingly, when Rps9a-GFP expression was examined in the single and double mutants, each double mutant restored full-length Rps9a-GFP levels back to WT (Fig. 8, *D* and *E*). Furthermore, none of the double mutants, including *vps27Δ not4Δ*, prevented Rps9a-GFP ribophagy. Instead, each increased conversion of the partial Rps9a-GFP degradation product in *not4Δ* to free GFP (Fig. 8, *D* and *E*), indicating that loss of these factors partially alleviates the block in complete 40S ribophagy due to *not4Δ*. Therefore, although WT cells require Vps27 for 40S ribophagy during TORC1 stress, redundant mechanisms must activate this process in *not4Δ*.

## Discussion

Cells coordinate anabolism and proliferation with their environment, which involves communication between the endolysosomal pathway, and the machinery controlling gene expression, translation, and proteostasis. Ccr4-Not regulates each of these processes, so it is uniquely positioned to integrate communication between these interrelated activities. While Ccr4-Not’s role in transcriptome regulation is known, how Ccr4-Not ubiquitin signaling affects the proteome remains incompletely understood. To address this issue, we performed proteomic analysis of cells lacking Not4, expressing WT Not4, or expressing a full-length inactive Not4 mutant. This approach controls for proteome alterations indirectly caused by Ccr4-Not structural changes that may affect its non-ligase signaling roles. Indeed, we find that complete Not4 loss decreases expression of the Caf40 and Not2 subunits, thus indicating that Not4 loss likely destabilizes other Ccr4-Not subunits as previously reported for other Ccr4-Not mutants (43). In addition, while *not4Δ* + vector and *not4Δ* + *NOT4RR* cells share many DEPs (422 in total) that represent proteins dependent on Not4 ligase signaling, *not4Δ* + vector cells also have ∼300 DEPs that are not explained due to the loss of Not4 ligase activity. These differences likely are explained through indirect effects on non-ligase dependent Ccr4-Not regulated activities due to destabilization of other Ccr4-Not subunits in the absence of Not4. Alternatively, Not4 may contribute to mRNA transcription, stability, and/or mRNA translation independently of its role as a ubiquitin ligase. We also find that increased Not4 levels upregulate mitochondrial proteins, indicating that Not4 ligase signaling may mediate mitochondrial metabolic reprogramming, although how this occurs remains unclear. Since Ccr4-Not controls multiple aspects of mitochondrial regulation (30, 39, 40, 75), future studies will need to address these important questions.

The two most intriguing findings presented herein are the RP and Rib repression due to dysregulated Ccr4-Not ligase activity, and the activation of 40S ribophagy when Ccr4-Not dependent ubiquitin signaling is lost. The repressed GOs for each experimental condition were dominated by ribosomal categories, clearly indicating that Not4 ligase signaling maintains ribosome homeostasis. These data are consistent with the previous report that Not4 loss inhibits polysome formation and alters ratios of 40S, 60S, and 80S ribosomes (24). Additionally, a previous metabolomic analysis found that the *not4Δ* metabolic profile is most similar to that of ribosomal mutants (76). These previous studies, and our data presented herein, provide increased support for the concept that Not4 ubiquitin signaling maintains ribosomal homeostasis. While Not4 overexpression restores many RP and Ribi factors, a core set remains repressed and overlaps those inhibited in both *not4Δ* + vector and *not4Δ* + *NOT4RR* cells (see Fig.S5 for visual representation). However, since Not4 overexpression corrects the protein synthesis defects and sensitivity to translational stress caused by *not4Δ*, their repression is insufficient to inhibit global protein synthesis. Another important observation is that the RP repression we detect is not selective solely for 40S or 60S RPs but it instead encompasses RPs from both. Our analysis of the model RP Rps9a indicates that neither proteasome nor macroautophagy inhibition restores Rps9a expression in *not4Δ*. Therefore, Not4 ligase activity does not inhibit RP expression by enhancing their degradation. Whether the mechanisms repressing both 40S and 60S RPs are the same or differ will need to be addressed in future studies, but it is clear this repression occurs post-transcriptionally.

We believe the most likely explanation for the RP and Ribi inhibitory effects is that Ccr4-Not ligase signaling affects RP and Ribi mRNA translation. Ccr4-Not binds to RP mRNAs during transcription elongation, which contributes to their cytoplasmic translation (43). If this process depends on Not4 ligase activity, then altering Not4 expression (too little or too much) and/or its function may impair RP mRNA translation. The RP inhibition detected also may be linked to the repression of specific Ribi factors that occurs since Ribi inhibition, perhaps due to defects in Ccr4-Not regulation of Pol I transcription (7), would be expected to cause ribosomal assembly defects (77). In this scenario, individual RPs and/or dysfunctionally assembled 40S ribosomes then could be flagged with ubiquitin to initiate their degradation *via* ribophagy. Altered Not4 levels and ligase-dependent inhibitory effects on RP expression also is supported by our targeted analysis of Rps9a and Rpl36a since Not4 expressed from its chromosomal regulatory elements restores their expression to wild-type levels. Increased Not4 levels in wild-type cells also enhance sensitivity to select translational stressors that impair ribosome activity. These genetic data suggest the possibility that factors that interact with the ribosome to affect tRNA-ribosome interactions may be particularly sensitive to Not4 ligase signaling. In total, these data support the concept that balanced Not4 ligase activity maintains ribosomal homeostasis. Future efforts will need to deconvolute the underlying mechanisms to discern how Not4 ligase signaling mediates this control.

Another key finding in this report is that either Not4 or Ccr4 loss activates 40S ribophagy, indicating they repress 40S ribophagy through a shared mechanism requiring Ccr4-Not ubiquitin signaling. Ccr4 regulates endolysosomal nutrient signaling independently of its deadenylase activity (30), while it also contributes to Not4-dependent ubiquitin signaling (55). As such, Ccr4 loss impairs a key aspect of Not4 ubiquitin signaling that prevents 40S ribophagy activation. This possibility is further supported by the results demonstrating that loss of the E2 enzymes Ubc4/Ubc5, which are not Ccr4-Not complex members, causes even greater partial 40S ribophagy than *not4Δ*. These data further demonstrate that Ccr4-Not 40S ribophagy activation is due specifically to the disruption of its ubiquitin ligase activity, and they suggest that additional E3 ligases function redundantly with Not4 to inhibit this process. While Ccr4-Not disruption deregulates macroautophagy in nutrient-replete conditions, macroautophagy cannot explain the 40S ribophagy since *atg7Δ not4Δ* fails to prevent it. Furthermore, both Rps9a-GFP repression and 40S ribophagy in *not4Δ* are not due solely to TORC1 inhibition since restoring TORC1 signaling in *not4Δ* has no effect on either process. Previously, we found that Ccr4-Not activates TORC1 by promoting vacuole V-ATPase function (30). Our unpublished data indicate that *not4Δ* has a greater inhibitory effect on V-ATPase-dependent nutrient signaling than *ccr4Δ*, so additional V-ATPase nutrient signaling pathways promoting RP expression may be impaired specifically in *not4Δ*. Similarly, the incomplete 40S ribophagy in *not4Δ* likely a consequence of this greater V-ATPase inhibition since the vacuole-specific proteolytic activities required for efficient 40S ribophagy require an acidic vacuole. This possibility is supported by our finding that loss of the plasma membrane pump Pdr5, or the vacuole Na^+^-K^+^/H^+^ exchanger Nhx1, both restores RP expression and enhances complete 40S ribophagy. These mutants likely compensate for the impaired V-ATPase activity in *not4Δ* by reducing competition for limiting intracellular H^+^ or by decreasing vacuole H^+^ efflux. Such an effect would decrease vacuole acidity, increase protein degradation, and promote the downstream vacuole-dependent nutrient signaling controlling RP expression.

Since the 40S ribophagy in *not4Δ* is independent of macroautophagy, it must result from the mechanistically distinct microautophagy pathway. K48 and K63 linked substrate polyubiquitination can signal degradation through both macroautophagy and microautophagy (22, 23). Our data indicates that K48-linked ubiquitin chains accumulate on Rps9a in *not4Δ*, suggesting this ubiquitination may flag the 40S for ribophagy similar to that which occurs for proteaphagy. How K48-linked 40S ubiquitination accumulates is not known, but it may be connected to the defects in proteasome-dependent deubiquitination that occur in Not4 mutants. Ribosome downregulation mediates adaptation to proteostatic stress (78), so the combined RP/Ribi repression and 40S ribophagy activation in *not4Δ* may allow adaptation to the increased proteostatic stress due to *not4Δ* proteosome dysregulation.

Three proteins previously connected to various autophagy pathways (Cue5, Nvj1, and Vps27) are upregulated in *not4Δ* vector and *NOT4RR* cells where 40S ribophagy is activated. Importantly, only loss of Vps27, which is an ESCRT ubiquitin binding subunit that is both required for vesicular transport and also is a microautophagy effector, blocked TORC1 stress-induced 40S ribophagy in wild-type cells. To our knowledge, this is the first factor identified to be required for 40S ribophagy. However, since 40S ribophagy was not prevented by the *vps27Δ not4Δ* mutant, redundant pathways must mediate 40S ribophagy in *not4Δ*. Surprisingly, the 40S RP degradation defect in *not4Δ* was partially restored by *vps27Δ not4Δ*, *cue5Δ not4Δ*, and *nvj1Δ not4Δ*. How co-disruption of these pathways enhances 40S RP degradation currently is not understood, but their effect appears similar to that which occurs upon loss of Pdr5 or Nhx1. A common theme linking them is their functional connection to endolysosomal-dependent nutrient signaling and stress pathways. Cue5 is a ubiquitin-binding receptor for proteaphagy and aggrephagy (68, 69), yet it has extensive genetic interactions with endolysosomal factors indicating it may have endolysosomal functions not yet identified (37, 79). Cue5 loss in *not4Δ* may alter these other activities to restore RP expression and enhance 40S ribophagy. Although Nvj1 mediates PMN, it also interacts with the oxysterol-binding protein Osh1 which promotes nutrient transport from the plasma membrane (71). A *nvj1Δ* enhances nutrient uptake by preventing sequestration of Osh1 from this additional role (80), so the *nvj1Δ not4Δ* may enhance nutrient signaling to compensate for the *not4Δ* defects. Since Vps27 also mediates vesicular transport, the *vps27Δ* may increase nutrient transporters on the cell surface as their turnover could be reduced through the endolysosomal pathway (18), which also would enhance endolysosomal nutrient signaling. Thus, the vacuole defects in *not4Δ* may be alleviated by the loss of these factors through multiple candidate mechanisms.

During the course of this study, a SILAC-based analysis of the WT and *not4Δ* proteome found that *not4Δ* increases the expression of several RPs relative to WT cells (42), which is a result seemingly at odds with our proteomic data. Previous studies using specialized protein extraction approaches found that *not4Δ* can form protein aggregates *in vivo*, which may be related to the loss of Rps7a monoubiquitination (24, 25). However, to prevent aggregates we prepared the proteomic samples by bead beading in a high salt buffer. We also directly demonstrate that both Rps9a and Rpl36a are repressed in *not4Δ* even when analyzed under denaturing conditions. Additionally, ribosomal protein aggregation would not account for the significant RP and Ribi repression in *not4Δ* + *NOT4* reconstituted cells where Rps7a monoubiquitination is intact, nor would it explain why disrupting endolysosomal effectors in *not4Δ* restores RP expression. Future efforts will be required to reconcile the differences between our results and this recent report.

Analysis of Ccr4-Not has focused on its role in transcription and mRNA decay. However, increasing evidence indicates that its ubiquitin signaling role is critically important to additional cellular processes. The data presented herein demonstrate that balanced Not4 ligase activity is required to maintain RP expression, while the Ccr4-Not ubiquitin ligase inhibits 40S ribophagy. Combined with our previous study connecting Ccr4-Not to endolysosomal regulation, these new data reveal increased importance for the Ccr4-Not ligase in endolysosomal-dependent nutrient signaling and autophagy control than currently is appreciated. Since Ccr4-Not dysregulation is implicated in many diseases and developmental disorders, probing these additional Ccr4-Not regulated processes will be essential to understanding Ccr4-Not’s role in these conditions.

## Experimental procedures

### Yeast strains and culture conditions

All strains in this study are derived from the BY4741 or DF5 genetic backgrounds and are listed in Table S1 (81). For all experiments, cells were cultured in an incubated shaker at 30 °C and harvested between OD_600_= 0.8 to 1.6. Cells were cultured in nutrient-rich YPD (1% yeast extract/2% peptone/2% dextrose). To select for plasmid transformants, cells were cultured in synthetic complete (SC) media containing 0.2% yeast nitrogen base without amino acids, 0.5% ammonium sulfate, 2% dextrose, and 0.19% amino acid dropout mix with amino acids or uracil added back except the specific one needed to maintain plasmid selection. For the puromycin labeling experiment, puromycin was added to the cultures at a final concentration of 10 μM and samples were taken at the indicated timepoints. The genetic manipulations used to either delete target genes or to engineer genomically integrated in-frame epitope tags, were performed as described (82). All yeast plasmids used in this study are listed in Table S2.

### Chemical reagents

Most chemicals, including rapamycin, Hygromycin B, and puromycin, were purchased either through Fisher or Millipore Sigma. Yeast media was purchased from US Biologicals and 5-Fluoroorotic acid (5-FOA) was purchased from Research Products International.

### RNA extraction and RT-qPCR analysis

Total RNA was prepared by hot acid phenol extraction from three independent biological replicates per experimental condition. The ImProm-II reverse transcription kit (Promega) was used to synthesize cDNA from 1 μg of total RNA using the included oligo dT primers in a volume of 20 μl per the manufacturer’s direction. After cDNA synthesis, the cDNA reaction was diluted with ultrapure dH_2_O to a final volume of 100 μl, and 1 μl of the diluted cDNA was used to perform gene-specific qPCR using SYBR Green reagent and an ABI StepOne Plus thermal cycler. The gene-specific signal was normalized to the *SPT15* internal reference control gene using the formula 2^(Ct*SPT15*-Ct*Target*)^ as previously described (83). The mean and standard deviation for each gene were calculated and are plotted in Figs. S3 and S7. Primer sequences used in this study are in Supplemental File 1. All statistical analyses and graphs were generated using GraphPad Prism 10.

### Antibodies and immunoblotting reagents

All immunoblots are representative of at least three independent experiments. The α-HA (clone F-7, catalog number sc7392), α-GFP (clone B-2, catalog number sc9996), and α-ubiquitin (clone P4D1, catalog number sc8017) antibodies were purchased from Santa Cruz Biotechnology. The α-Rps3 antibody was purchased from Proteintech (catalog number 11990-1-AP), while the α-FLAG (clone M2, catalog number F1804) and α-glucose-6-phosphate dehydrogenase (G6PDH, catalog number A9521) antibodies were purchased from Millipore Sigma. The α-puromycin antibody (clone PMY-2A4) was purchased from the Developmental Hybridoma Studies bank. Mouse and rabbit HRP-conjugated light chain-specific secondary antibodies were purchased from Jackson ImmunoResearch Laboratories (catalog numbers 115-035-174 and 211-032-171). Immunoblots were performed using MilliporeSigma Immobilon Western Chemiluminescent HRP Substrate and images were quantified using ImageJ.

### Whole-cell extract preparation

Whole-cell extracts (WCEs) were prepared by bead beating in extraction buffer (300 mM NaCl, 10 mM Tris, pH 8.0, 0.1% NP-40, 10% glycerol) and containing protease inhibitors (2 μg/ml each of aprotinin, pepstatin, and leupeptin), phosphatase inhibitor cocktail set II (Millipore Sigma, 1:100 dilution) and 1 mM DTT. Cell lysates were clarified for 15′ at 15,000 rpm @ 4 °C, supernatants then were transferred to a new tube, and the protein concentration was determined by Bradford assay.

### TUBE ubiquitin linkage analysis

WT and *not4Δ* Rps9a-GFP expressing cells were cultured to log phase in YPD media, harvested, and WCEs then prepared by bead beating in extraction buffer (150 mM NaCl, 10 mM Tris, pH 8.0, 0.1% NP-40, 10% glycerol, and 60 mM 2-chloroacetamide) containing protease inhibitors (2 μg/ml each of aprotinin, pepstatin, and leupeptin), and phosphatase inhibitor cocktail set II (Millipore Sigma, 1:100 dilution). For each sample, 600 μg of WCE was mixed with 2 μg of α-FLAG antibody in a total volume of 400 μl WCE buffer that had been mock treated or treated with 3 μg of K48ub or K63ub linkage specific Tandem Ubiquitin Binding Entities (TUBEs) purchased from LifeSensors. Samples were rotated at 4 °C for a minimum of 2 h before adding Protein A agarose beads and rotating at 4 °C for an additional 1 h to capture the immune complexes. Samples were washed three times with buffer and then resolved by 10% SDS-PAGE and analyzed by α-GFP immunoblotting.

### Confocal microscopy

The procedure for fluorescence microscopy was adopted from methods previously described with slight modifications (84, 85). The Rps9a-GFP wild-type strain was grown in 5 ml SC media overnight with shaking at 30 °C. In the morning, 1 ml SC media was inoculated such that the initial OD_600_ was approximately 0.1. Immediately after inoculation, 1 μl FM4-64 dye (from 1 mg/ml stock in DMSO from Invitrogen) was added to the culture, and tubes were incubated as above. After 5 to 6 h, cells were pelleted by centrifuging at 5000*g* for 5 min, washed twice with PBS pH7.4, and the cell pellet then was resuspended in a small volume of PBS pH7.4. A 5 μl cell suspension was placed on a glass slide, mixed with 5 μl of 1% low melting agarose (maintained in a 37 °C water bath), covered with a cover slip, and images were captured immediately using a Zeiss LSM 700 confocal microscope with a 63× objective. Image analysis was performed using Zeiss Zen Blue software.

### Tandem mass tag labeling and quantitative proteomic analysis

WCEs were prepared in 300 mM NaCl lysis buffer as described above. Samples (50ul, 50ug protein) then were reduced with 5 mM DTT for 45 min at 50 °C, alkylated with 25 mM iodoacetamide for 20′ at room temperature in the dark, and then incubated with 20 mM DTT for an additional 15′ at room temperature. Samples then were precipitated with five volumes of cold acetone (at −20 °C overnight), and proteins were pelleted at 16,000*g* at 4 °C for 10′. Protein pellets were washed with 100 μl of cold (−20 °C) 90% acetone, air dried for 4 minutes, and re-dissolved in 100 μl of digestion buffer (100 mM HEPES, pH 8.3) containing 0.5 μg of Lys-C enzyme (Waco, Fuji, 125-05061). The protein mix then was digested for 2 h at 37 °C with shaking; adding 1ug trypsin (Thermo Fisher, 900057), the digestion was continued overnight.

For TMTpro-16plex labeling, a set of 16 samples each containing 50 μg of peptides in 100 μl of digestion buffer (100 mm HEPES pH 8.3) was labeled using a commercial TMTpro-16plex Mass Tag Labeling reagent kit (A44521, Thermo Fisher) according to the manufacturer’s protocol. The labeled set of 16 samples was combined, vacuum-dried, and reconstituted in 0.1% TFA at 0.3 μg/μl for further fractionation. 300 μl (90 μg) of reconstituted mixture of labeled peptides was fractionated using Pierce High pH Reversed-Phase Peptide Fractionation kit (84,868, Thermo Fisher) according to the manufacturer’s protocol for TMT-labeled peptides, with eight-step fractions (consecutively eluted with 10.0, 12.5, 15.0, 17.5, 20.0, 22.5, 25.0, and 50.0% acetonitrile) collected. The peptide fractions were vacuum dried, dissolved in 65 μl of loading buffer (3% ACN with 0.1% TFA), and 5 μl (0.87 μg, assuming elution of equal amounts of peptides per fraction) aliquots were analyzed by LC-MS for peptide/protein identification and quantification.

Acquisition of raw MS data was performed on an Orbitrap Fusion Lumos mass spectrometer (Thermo Fisher) operating in line with Ultimate 3000RSLCnano UHPLS system (Thermo Fisher) using MS2 method for TMTpro-16plex labeled samples with 160 min LC gradient. The peptides were trapped on an Acclaim PepMap 100 nanoViper column (75 μm × 20 mm, Thermo Fisher) at 5 μl/min flow rate and washed with loading buffer for 5 min. The trapped peptides were separated on an Acclaim PepMap RSLC nanoViper column (75 μm × 500 mm, C-18, 2 μm, 100 Å, Thermo Fisher) at 300 nl/min flow rate and 40 °C column temperature using water and acetonitrile with 0.1% formic acid as solvents A and B, respectively. The following multi-point linear gradient was applied: 3% B at 0-4 min, 5% B at 5′, 23% B at 110′, 30% B at 120′, 90% B at 123 to 133′, 3% B at 136 to 160′. MS2 acquisition method was used with three-second cycles with the following MS scan parameters. Full MS scans were performed in the Orbitrap analyzer at 120,000 (FWHM, at m/z = 200) resolving power to determine the accurate masses (m/z) of peptides. Following MS1 full scans, data-dependent MS2 scans were performed on precursor ions with peptide isotopic pattern, charge state 2 to 6, and intensity of at least 25,000. Peptide ions were isolated in the quadrupole with 0.7 m/z window, fragmented (HCD, 38% NCE), and the masses/intensities of fragment and reporter ions were determined with an Orbitrap analyzer at 50,000 (FWHM, at m/z = 200) resolving power and dynamic exclusion applied for 45 s.

Post-acquisition analysis of raw MS data was performed within a mass informatics platform Proteome Discoverer 2.4 (Thermo Fisher) using Sequest HT search algorithm and yeast protein database (SwissProt, *Saccharomyces cerevisiae* S288c, TaxID 559292, v.2017-10-25, 6727 entries). The reversed target database was used as decoy database. The raw MS data acquired for the set of eight fractions (derived from the same mixture of labeled samples) were treated as ‘Fractions’ for post-acquisition analysis. Full tryptic peptides were searched and two mis-cleavages were allowed. The searched fixed modifications included: carbamidomethylation of Cys residues and TMTpro modification of Lys residues and any peptide N-terminus. The variable modifications included oxidation of Met residues, acetylation, and Met-loss of the protein N-terminus. The precursor, fragment, and reporter ion mass tolerances were set to 10 ppm, 0.02 Da, and 20 ppm, respectively. The raw data were filtered for the precursor ions with S/N of at least 1.5. The PSMs were filtered for further analysis using a delta Cn threshold of 0.05. The q-values were calculated at PSM level (Percolator) and then, at the peptide level (Qvality algorithm), to control for the false discovery rate (FDR). The FDR threshold of 0.01 was used to validate and filter the PSMs and the corresponding peptide sequences. The validated/filtered peptides were used for the identification of the candidate precursor proteins. Each candidate precursor protein was scored by summing the PEP values of the assigned peptides. The sum-PEP protein scores were used to calculate the experimental q-values at the protein level. The candidate proteins were further validated using 0.01 (strict) and 0.05 (relaxed) FDR thresholds. A set of candidate proteins was accepted as an identified protein group if none of the assigned peptides was unique to any protein, but at least one of those peptides was common and unique to that group of proteins. Strict parsimony principle was applied to the protein groups. The reporter ions quantification values (based on S/N ratio) were corrected according to the product data sheet provided with the used TMTpro-16plex Label reagent set. The co-isolation and average S/N ratio thresholds were set to 50% and 10, respectively. Unique and razor peptides were used for protein quantification, and protein groups were considered for peptide uniqueness.

### Proteome data analysis

The data generated by the mass spectrometry analysis was transferred to the UTHSC Molecule Bioinformatics Core using SFTP. These data then were normalized using the normalizeCylclicLoess function from R/Bioconductor-package limma (86). The normalized data matrix was loaded into R, and statistics were gathered and used to determine differential expression. The mean, variance, standard deviation, and standard error of means were calculated for each protein across the different conditions. Pearson’s correlation coefficient was graphed in order to identify sample outliers in each condition. At this time, no outliers were determined. A principal component analysis was performed to determine different clusters in the samples, and fold change was then calculated for all proteins. The significance for each protein was assessed by using one-way ANOVA, and the *p* values were adjusted for multiplicity using the Benjamini-Hochberg method (87). Only proteins with an adjusted *p* value < 0.05 were considered differentially expressed (File S2). These proteins are used to create a heatmap with unsupervised hierarchical clustering. The protein list then was loaded into the STRING database for protein-protein interaction mapping and to test for enriched cellular component GO categories (44).

## Data availability

The mass spectrometry proteomics data have been deposited to the ProteomeXchange Consortium *via* the PRIDE partner repository with the dataset identifier PXD044420 and 10.6019/PXD044420" (88, 89). Submission details for accessing the dataset are as follows:

**Project Name:** Ccr4-Not ubiquitin ligase signaling regulates ribosomal protein homeostasis and inhibits 40S ribosomal autophagy.

**Project accession:**PXD044420.

**Project DOI:**10.6019/PXD044420.

Reviewer account details:

**Username:**reviewer_pxd044420@ebi.ac.uk.

**Password:** 4tCDgHXm.

## Supporting information

This article contains supporting information (11, 66, 90, 91, 92).

## Conflict of interest

The authors declare that they have no conflicts of interest with the contents of this article.

## Supporting information

File S3

Siupplemental Figure

## References

[1] Jain A., Zoncu R. (2022). Organelle transporters and inter-organelle communication as drivers of metabolic regulation and cellular homeostasis. Mol. Metab..

[2] Liu G.Y., Sabatini D.M. (2020). mTOR at the nexus of nutrition, growth, ageing and disease. Nat. Rev. Mol. Cell Biol..

[3] Collart M.A. (2016). The Ccr4-Not complex is a key regulator of eukaryotic gene expression. Wiley Inter. Rev. RNA.

[4] Pavanello L., Hall M., Winkler G.S. (2023). Regulation of eukaryotic mRNA deadenylation and degradation by the Ccr4-Not complex. Front. Cell Dev. Biol..

[5] Kruk J.A., Dutta A., Fu J., Gilmour D.S., Reese J.C. (2011). The multifunctional Ccr4-Not complex directly promotes transcription elongation. Genes Dev..

[6] Kerr S.C., Azzouz N., Fuchs S.M., Collart M.A., Strahl B.D., Corbett A.H. (2011). The Ccr4-Not complex interacts with the mRNA export machinery. PLoS One.

[7] Laribee R.N., Hosni-Ahmed A., Workman J.J., Chen H. (2015). Ccr4-not regulates RNA polymerase I transcription and couples nutrient signaling to the control of ribosomal RNA biogenesis. PLoS Genet..

[8] Mulder K.W., Inagaki A., Cameroni E., Mousson F., Winkler G.S., De Virgilio C. (2007). Modulation of Ubc4p/Ubc5p-mediated stress responses by the RING-finger-dependent ubiquitin-protein ligase Not4p in Saccharomyces cerevisiae. Genetics.

[9] Mersman D.P., Du H.N., Fingerman I.M., South P.F., Briggs S.D. (2009). Polyubiquitination of the demethylase Jhd2 controls histone methylation and gene expression. Genes Dev..

[10] Cooper K.F., Scarnati M.S., Krasley E., Mallory M.J., Jin C., Law M.J. (2012). Oxidative-stress-induced nuclear to cytoplasmic relocalization is required for Not4-dependent cyclin C destruction. J. Cell Sci..

[11] Chen H., Sirupangi T., Wu Z.H., Johnson D.L., Laribee R.N. (2018). The conserved RNA recognition motif and C3H1 domain of the Not4 ubiquitin ligase regulate *in vivo* ligase function. Sci. Rep..

[12] Allen G., Weiss B., Panasenko O.O., Huch S., Villanyi Z., Albert B. (2023). Not1 and Not4 inversely determine mRNA solubility that sets the dynamics of co-translational events. Genome Biol..

[13] Cano F., Miranda-Saavedra D., Lehner P.J. (2010). RNA-binding E3 ubiquitin ligases: novel players in nucleic acid regulation. Biochem. Soc. Trans..

[14] Jiang H., Wolgast M., Beebe L.M., Reese J.C. (2019). Ccr4-Not maintains genomic integrity by controlling the ubiquitylation and degradation of arrested RNAPII. Genes Dev..

[15] Domanska A., Kaminska J. (2015). Role of Rsp5 ubiquitin ligase in biogenesis of rRNA, Mrna Trna yeast. RNA Biol..

[16] Rodriguez M.S., Gwizdek C., Haguenauer-Tsapis R., Dargemont C. (2003). The HECT ubiquitin ligase Rsp5p is required for proper nuclear export of mRNA in Saccharomyces cerevisiae. Traffic.

[17] Neumann S., Petfalski E., Brugger B., Grosshans H., Wieland F., Tollervey D. (2003). Formation and nuclear export of tRNA, rRNA and mRNA is regulated by the ubiquitin ligase Rsp5p. EMBO Rep..

[18] Babst M. (2014). Quality control: quality control at the plasma membrane: one mechanism does not fit all. J. Cell Biol..

[19] Kraft C., Peter M. (2008). Is the Rsp5 ubiquitin ligase involved in the regulation of ribophagy?. Autophagy.

[20] Belgareh-Touze N., Cavellini L., Cohen M.M. (2017). Ubiquitination of ERMES components by the E3 ligase Rsp5 is involved in mitophagy. Autophagy.

[21] Yang X., Zhang W., Wen X., Bulinski P.J., Chomchai D.A., Arines F.M. (2020). TORC1 regulates vacuole membrane composition through ubiquitin- and ESCRT-dependent microautophagy. J. Cell Biol..

[22] Li J., Hochstrasser M. (2022). Selective microautophagy of proteasomes is initiated by ESCRT-0 and is promoted by proteasome ubiquitylation. J. Cell Sci..

[23] Marshall R.S., Vierstra R.D. (2022). A trio of ubiquitin ligases sequentially drives ubiquitylation and autophagic degradation of dysfunctional yeast proteasomes. Cell Rep..

[24] Panasenko O.O., Collart M.A. (2012). Presence of Not5 and ubiquitinated Rps7A in polysome fractions depends upon the Not4 E3 ligase. Mol. Microbiol..

[25] Preissler S., Reuther J., Koch M., Scior A., Bruderek M., Frickey T. (2015). Not4-dependent translational repression is important for cellular protein homeostasis in yeast. EMBO J..

[26] Panasenko O., Landrieux E., Feuermann M., Finka A., Paquet N., Collart M.A. (2006). The yeast Ccr4-Not complex controls ubiquitination of the nascent-associated polypeptide (NAC-EGD) complex. J. Biol. Chem..

[27] Panasenko O.O., Collart M.A. (2011). Not4 E3 ligase contributes to proteasome assembly and functional integrity in part through Ecm29. Mol. Cell Biol..

[28] Fu X., Sokolova V., Webb K.J., Old W., Park S. (2018). Ubiquitin-dependent switch during assembly of the proteasomal ATPases mediated by Not4 ubiquitin ligase. Proc. Natl. Acad. Sci. U. S. A..

[29] Nahar A., Sokolova V., Sekaran S., Orth J.D., Park S. (2022). Assembly checkpoint of the proteasome regulatory particle is activated by coordinated actions of proteasomal ATPase chaperones. Cell Rep..

[30] Chen H., Miller P.W., Johnson D.L., Laribee R.N. (2020). The Ccr4-Not complex regulates TORC1 signaling and mitochondrial metabolism by promoting vacuole V-ATPase activity. PLoS Genet..

[31] Buschauer R., Matsuo Y., Sugiyama T., Chen Y.H., Alhusaini N., Sweet T. (2020). The Ccr4-Not complex monitors the translating ribosome for codon optimality. Science.

[32] Yin Z., Zhang Z., Lei Y., Klionsky D.J. (2023). Bidirectional roles of the Ccr4-Not complex in regulating autophagy before and after nitrogen starvation. Autophagy.

[33] Dechant R., Saad S., Ibanez A.J., Peter M. (2014). Cytosolic pH regulates cell growth through distinct GTPases, Arf1 and Gtr1, to promote Ras/PKA and TORC1 activity. Mol. Cell.

[34] Dechant R., Binda M., Lee S.S., Pelet S., Winderickx J., Peter M. (2010). Cytosolic pH is a second messenger for glucose and regulates the PKA pathway through V-ATPase. EMBO J..

[35] Zoncu R., Bar-Peled L., Efeyan A., Wang S., Sancak Y., Sabatini D.M. (2011). mTORC1 senses lysosomal amino acids through an inside-out mechanism that requires the vacuolar H(+)-ATPase. Science.

[36] Pan X., Ye P., Yuan D.S., Wang X., Bader J.S., Boeke J.D. (2006). A DNA integrity network in the yeast Saccharomyces cerevisiae. Cell.

[37] Costanzo M., VanderSluis B., Koch E.N., Baryshnikova A., Pons C., Tan G. (2016). A global genetic interaction network maps a wiring diagram of cellular function. Science.

[38] Costanzo M., Baryshnikova A., Bellay J., Kim Y., Spear E.D., Sevier C.S. (2010). The genetic landscape of a cell. Science.

[39] Azzouz N., Panasenko O.O., Deluen C., Hsieh J., Theiler G., Collart M.A. (2009). Specific roles for the Ccr4-Not complex subunits in expression of the genome. RNA.

[40] Cui Y., Ramnarain D.B., Chiang Y.C., Ding L.H., McMahon J.S., Denis C.L. (2008). Genome wide expression analysis of the CCR4-NOT complex indicates that it consists of three modules with the NOT module controlling SAGA-responsive genes. Mol. Genet. Genomics.

[41] Miller J.E., Zhang L., Jiang H., Li Y., Pugh B.F., Reese J.C. (2018). Genome-Wide mapping of decay factor-mRNA interactions in yeast identifies nutrient-responsive transcripts as targets of the Deadenylase Ccr4. G3 (Bethesda).

[42] Allen G.E., Panasenko O.O., Villanyi Z., Zagatti M., Weiss B., Pagliazzo L. (2021). Not4 and Not5 modulate translation elongation by Rps7A ubiquitination, Rli1 moonlighting, condensates that exclude Eif5a. Cell Rep..

[43] Gupta I., Villanyi Z., Kassem S., Hughes C., Panasenko O.O., Steinmetz L.M. (2016). Translational capacity of a cell is determined during transcription elongation *via* the ccr4-not complex. Cell Rep..

[44] Szklarczyk D., Morris J.H., Cook H., Kuhn M., Wyder S., Simonovic M. (2017). The STRING database in 2017: quality-controlled protein-protein association networks, made broadly accessible. Nucleic Acids Res..

[45] Broach J.R. (2012). Nutritional control of growth and development in yeast. Genetics.

[46] Kelleher A.R., Kimball S.R., Dennis M.D., Schilder R.J., Jefferson L.S. (2013). The mTORC1 signaling repressors REDD1/2 are rapidly induced and activation of p70S6K1 by leucine is defective in skeletal muscle of an immobilized rat hindlimb. Am. J. Physiol. Endocrinol. Metab..

[47] Brodersen D.E., Clemons W.M., Carter A.P., Morgan-Warren R.J., Wimberly B.T., Ramakrishnan V. (2000). The structural basis for the action of the antibiotics tetracycline, pactamycin, and hygromycin B on the 30S ribosomal subunit. Cell.

[48] Schneider-Poetsch T., Ju J., Eyler D.E., Dang Y., Bhat S., Merrick W.C. (2010). Inhibition of eukaryotic translation elongation by cycloheximide and lactimidomycin. Nat. Chem. Biol..

[49] Vijayraghavan U., Company M., Abelson J. (1989). Isolation and characterization of pre-mRNA splicing mutants of Saccharomyces cerevisiae. Genes Dev..

[50] Klionsky D.J. (2011). For the last time, it is GFP-Atg8, not Atg8-GFP (and the same goes for LC3). Autophagy.

[51] An H., Harper J.W. (2020). Ribosome abundance control *via* the ubiquitin-proteasome system and autophagy. J. Mol. Biol..

[52] Sung M.K., Reitsma J.M., Sweredoski M.J., Hess S., Deshaies R.J. (2016). Ribosomal proteins produced in excess are degraded by the ubiquitin-proteasome system. Mol. Biol. Cell.

[53] Sung M.K., Porras-Yakushi T.R., Reitsma J.M., Huber F.M., Sweredoski M.J., Hoelz A. (2016). A conserved quality-control pathway that mediates degradation of unassembled ribosomal proteins. eLife.

[54] Hjerpe R., Aillet F., Lopitz-Otsoa F., Lang V., England P., Rodriguez M.S. (2009). Efficient protection and isolation of ubiquitylated proteins using tandem ubiquitin-binding entities. EMBO Rep..

[55] Kandasamy G., Pradhan A.K., Palanimurugan R. (2021). Ccr4-Not complex subunits Ccr4, Caf1, and Not4 are novel proteolysis factors promoting the degradation of ubiquitin-dependent substrates by the 26S proteasome. Biochim. Biophys. Acta Mol. Cell Res..

[56] Finley D., Ulrich H.D., Sommer T., Kaiser P. (2012). The ubiquitin-proteasome system of Saccharomyces cerevisiae. Genetics.

[57] Wagner M., Blum D., Raschka S.L., Nentwig L.M., Gertzen C.G.W., Chen M. (2022). A new twist in ABC transporter mediated multidrug resistance - pdr5 is a drug/proton Co-transporter. J. Mol. Biol..

[58] Collins G.A., Gomez T.A., Deshaies R.J., Tansey W.P. (2010). Combined chemical and genetic approach to inhibit proteolysis by the proteasome. Yeast.

[59] Shin H.R., Zoncu R. (2020). The lysosome at the intersection of cellular growth and destruction. Dev. Cell.

[60] Hirai H., Ohta K. (2023). Comparative Research: regulatory mechanisms of ribosomal gene transcription in Saccharomyces cerevisiae and Schizosaccharomyces pombe. Biomolecules.

[61] Orij R., Brul S., Smits G.J. (2011). Intracellular pH is a tightly controlled signal in yeast. Biochim. Biophys. Acta.

[62] Brett C.L., Tukaye D.N., Mukherjee S., Rao R. (2005). The yeast endosomal Na+K+/H+ exchanger Nhx1 regulates cellular pH to control vesicle trafficking. Mol. Biol. Cell.

[63] Schrezenmeier E., Dorner T. (2020). Mechanisms of action of hydroxychloroquine and chloroquine: implications for rheumatology. Nat. Rev. Rheumatol..

[64] Shao E., Forgac M. (2004). Involvement of the nonhomologous region of subunit A of the yeast V-ATPase in coupling and *in vivo* dissociation. J. Biol. Chem..

[65] Takeshige K., Baba M., Tsuboi S., Noda T., Ohsumi Y. (1992). Autophagy in yeast demonstrated with proteinase-deficient mutants and conditions for its induction. J. Cell Biol..

[66] Reinke A., Chen J.C., Aronova S., Powers T. (2006). Caffeine targets TOR complex I and provides evidence for a regulatory link between the FRB and kinase domains of Tor1p. J. Biol. Chem..

[67] Kraft C., Deplazes A., Sohrmann M., Peter M. (2008). Mature ribosomes are selectively degraded upon starvation by an autophagy pathway requiring the Ubp3p/Bre5p ubiquitin protease. Nat. Cell Biol..

[68] Lu K., Psakhye I., Jentsch S. (2014). Autophagic clearance of polyQ proteins mediated by ubiquitin-Atg8 adaptors of the conserved CUET protein family. Cell.

[69] Lu K., den Brave F., Jentsch S. (2017). Receptor oligomerization guides pathway choice between proteasomal and autophagic degradation. Nat. Cell Biol..

[70] Marshall R.S., McLoughlin F., Vierstra R.D. (2016). Autophagic turnover of inactive 26S proteasomes in yeast is directed by the ubiquitin receptor Cue5 and the Hsp42 chaperone. Cell Rep..

[71] Kvam E., Goldfarb D.S. (2004). Nvj1p is the outer-nuclear-membrane receptor for oxysterol-binding protein homolog Osh1p in Saccharomyces cerevisiae. J. Cell Sci..

[72] Oku M., Maeda Y., Kagohashi Y., Kondo T., Yamada M., Fujimoto T. (2017). Evidence for ESCRT- and clathrin-dependent microautophagy. J. Cell Biol..

[73] Raiborg C., Rusten T.E., Stenmark H. (2003). Protein sorting into multivesicular endosomes. Curr. Opin. Cell Biol..

[74] Ossareh-Nazari B., Nino C.A., Bengtson M.H., Lee J.W., Joazeiro C.A., Dargemont C. (2014). Ubiquitylation by the Ltn1 E3 ligase protects 60S ribosomes from starvation-induced selective autophagy. J. Cell Biol..

[75] Wu Z., Wang Y., Lim J., Liu B., Li Y., Vartak R. (2018). Ubiquitination of ABCE1 by NOT4 in response to mitochondrial damage links Co-translational quality control to PINK1-directed mitophagy. Cell Metab.

[76] Mulleder M., Calvani E., Alam M.T., Wang R.K., Eckerstorfer F., Zelezniak A. (2016). Functional metabolomics describes the yeast biosynthetic regulome. Cell.

[77] Woolford J.L., Baserga S.J. (2013). Ribosome biogenesis in the yeast Saccharomyces cerevisiae. Genetics.

[78] Guerra-Moreno A., Isasa M., Bhanu M.K., Waterman D.P., Eapen V.V., Gygi S.P. (2015). Proteomic analysis identifies ribosome reduction as an effective proteotoxic stress response. J. Biol. Chem..

[79] Schuldiner M., Collins S.R., Thompson N.J., Denic V., Bhamidipati A., Punna T. (2005). Exploration of the function and organization of the yeast early secretory pathway through an epistatic miniarray profile. Cell.

[80] Kvam E., Goldfarb D.S. (2006). Structure and function of nucleus-vacuole junctions: outer-nuclear-membrane targeting of Nvj1p and a role in tryptophan uptake. J. Cell Sci..

[81] Chen P., Johnson P., Sommer T., Jentsch S., Hochstrasser M. (1993). Multiple ubiquitin-conjugating enzymes participate in the *in vivo* degradation of the yeast MAT alpha 2 repressor. Cell.

[82] Janke C., Magiera M.M., Rathfelder N., Taxis C., Reber S., Maekawa H. (2004). A versatile toolbox for PCR-based tagging of yeast genes: new fluorescent proteins, more markers and promoter substitution cassettes. Yeast.

[83] Chen H., Fan M., Pfeffer L.M., Laribee R.N. (2012). The histone H3 lysine 56 acetylation pathway is regulated by target of rapamycin (TOR) signaling and functions directly in ribosomal RNA biogenesis. Nucleic Acids Res..

[84] Kumar R., Shroff A., Nazarko T.Y. (2022). Komagataella phaffii Cue5 piggybacks on lipid droplets for its vacuolar degradation during stationary phase lipophagy. Cells.

[85] Boutouja F., Stiehm C.M., Reidick C., Mastalski T., Brinkmeier R., Magraoui F.E. (2019). Vac8 controls vacuolar membrane dynamics during different autophagy pathways in Saccharomyces cerevisiae. Cells.

[86] Ritchie M.E., Phipson B., Wu D., Hu Y., Law C.W., Shi W. (2015). Limma powers differential expression analyses for RNA-sequencing and microarray studies. Nucleic Acids Res..

[87] Benjamini Y., Hochberg Y. (1995). Controlling the false discovery rate: a practical and powerful approach to multiple testing. J. Royal Statist Soc. Ser. B.

[88] Deutsch E.W., Bandeira N., Perez-Riverol Y., Sharma V., Carver J.J., Mendoza L. (2023). The ProteomeXchange consortium at 10 years: 2023 update. Nucleic Acids Res..

[89] Perez-Riverol Y., Bai J., Bandla C., Garcia-Seisdedos D., Hewapathirana S., Kamatchinathan S. (2022). The PRIDE database resources in 2022: a hub for mass spectrometry-based proteomics evidences. Nucleic Acids Res..

[90] Mumberg D., Muller R., Funk M. (1995). Yeast vectors for the controlled expression of heterologous proteins in different genetic backgrounds. Gene.

[91] Sikorski R.S., Hieter P. (1989). A system of shuttle vectors and yeast host strains designed for efficient manipulation of DNA in Saccharomyces cerevisiae. Genetics.

[92] Guan J., Stromhaug P.E., George M.D., Habibzadegah-Tari P., Bevan A., Dunn W.A. (2001). Cvt18/Gsa12 is required for cytoplasm-to-vacuole transport, pexophagy, and autophagy in Saccharomyces cerevisiae and Pichia pastoris. Mol. Biol. Cell.

