# Supplementary material for: Ccr4-not ubiquitin ligase signaling regulates ribosomal protein homeostasis and inhibits 40S ribosomal autophagy
Source: J Biol Chem. 2024 Jul 16;300(8):107582. doi: 10.1016/j.jbc.2024.107582 (PMC11357857; doi:10.1016/j.jbc.2024.107582)
Supplement: Siupplemental Figure [file mmc4.pdf]

Ccr4-Not ubiquitin ligase signaling regulates ribosomal protein homeostasis and inhibits 40S ribosomal autophagy

Daniel L. Johnson[#1], Ravinder Kumar[#2], David Kakhniashvili[3], Lawrence M. Pfeffer[2], and R. Nicholas Laribee[2*]

[1]- Molecular Bioinformatics Core and the University of Tennessee Health Science Center Office of Research, University of Tennessee Health Science Center, Memphis, TN, United States of America

[2]- Department of Pathology and Laboratory Medicine, College of Medicine and the Center for Cancer Research, University of Tennessee Health Science Center, Memphis, TN, United States of America.

[3]- Proteomics and Metabolomics Core and the University of Tennessee Health Science Center Office of Research, University of Tennessee Health Science Center, Memphis, TN, United States of America

[#]- These authors contributed equally.

[*]-Corresponding author:

R. Nicholas Laribee,

University of

Tennessee,

Department of Pathology and Laboratory

Medicine, Cancer Research Building

19 South Manassas, Room 318

Memphis, TN 38163

Phone: (901) 448-2609

Email: rlaribee@uthsc.edu

Figure S1. Proteomic quantification of Ccr4-Not subunit expression. The mean and SD of the normalized protein abundance for each indicated Ccr4-Not subunit is plotted. The expression of Caf40 and Not2 was reduced greater than 1.5-fold in the *not4Δ* + vector control relative to the WT + vector control. The expression difference for the remaining Ccr4-Not subunits across the different experimental conditions was less than 1.5-fold. The significance of the individual replicates between each condition was analyzed by one way ANOVA. *-$p<0.05$ or greater.

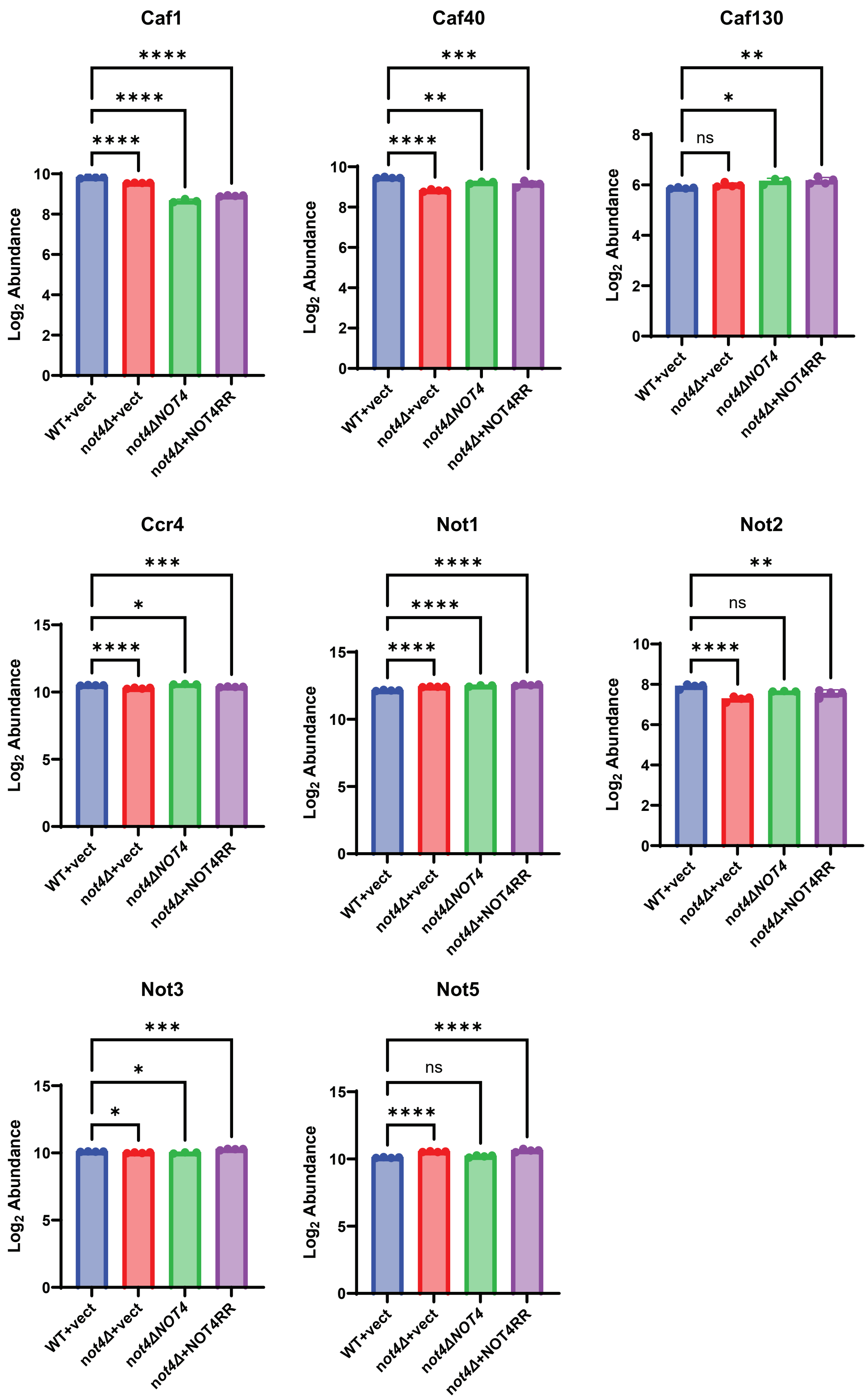
Caf1
Caf40
Caf130
Ccr4
Not1
Not2
Not3
Not5
Log2 Abundance
WT+vect
not4Δ+vect
not4ΔNOT4
not4Δ+NOT4RR
****
***
**
*
ns

Figure S1

Figure S2. DEP pathway enrichment analysis of WT + vector control relative to *not4Δ* + vector, *not4Δ* + *NOT4*, and *not4Δ* + *NOT4RR* expressing cells. Bubble plot analysis of the cellular component GO categories overrepresented in the *not4Δ* + vector down (**A**) and up (**B**) DEPs, the *not4Δ* + *NOT4* down (**C**) and up (**D**) DEPs, and the *not4Δ* + *NOT4RR* down (**E**) and up (**F**) DEPs.

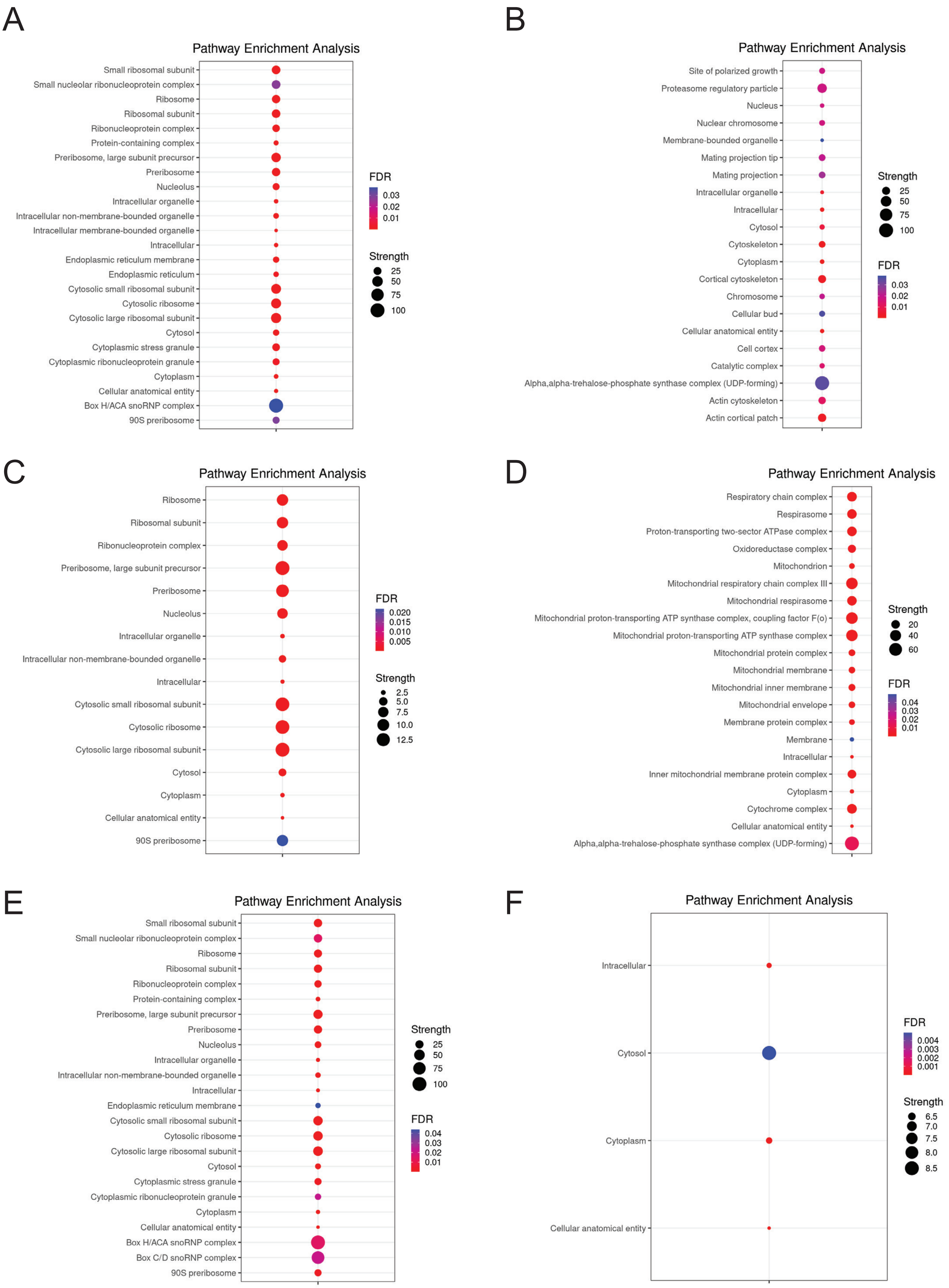
A
Pathway Enrichment Analysis
Small ribosomal subunit
Small nucleolar ribonucleoprotein complex
Ribosome
Ribosomal subunit
Ribonucleoprotein complex
Protein-containing complex
Preribosome, large subunit precursor
Preribosome
Nucleolus
Intracellular organelle
Intracellular non-membrane-bounded organelle
Intracellular membrane-bounded organelle
Intracellular
Endoplasmic reticulum membrane
Endoplasmic reticulum
Cytosolic small ribosomal subunit
Cytosolic ribosome
Cytosolic large ribosomal subunit
Cytosol
Cytoplasmic stress granule
Cytoplasmic ribonucleoprotein granule
Cytoplasm
Cellular anatomical entity
Box H/ACA snoRNP complex
90S preribosome
FDR
0.03
0.02
0.01
Strength
25
50
75
100
B
Pathway Enrichment Analysis
Site of polarized growth
Proteasome regulatory particle
Nucleus
Nuclear chromosome
Membrane-bounded organelle
Mating projection tip
Mating projection
Intracellular organelle
Intracellular
Cytosol
Cytoskeleton
Cytoplasm
Cortical cytoskeleton
Chromosome
Cellular bud
Cellular anatomical entity
Cell cortex
Catalytic complex
Alpha,alpha-trehalose-phosphate synthase complex (UDP-forming)
Actin cytoskeleton
Actin cortical patch
Strength
25
50
75
100
FDR
0.03
0.02
0.01
C
Pathway Enrichment Analysis
Ribosome
Ribosomal subunit
Ribonucleoprotein complex
Preribosome, large subunit precursor
Preribosome
Nucleolus
Intracellular organelle
Intracellular non-membrane-bounded organelle
Intracellular
Cytosolic small ribosomal subunit
Cytosolic ribosome
Cytosolic large ribosomal subunit
Cytosol
Cytoplasm
Cellular anatomical entity
90S preribosome
FDR
0.020
0.015
0.010
0.005
Strength
2.5
5.0
7.5
10.0
12.5
D
Pathway Enrichment Analysis
Respiratory chain complex
Respirasome
Proton-transporting two-sector ATPase complex
Oxidoreductase complex
Mitochondrion
Mitochondrial respiratory chain complex III
Mitochondrial respirasome
Mitochondrial proton-transporting ATP synthase complex, coupling factor F(o)
Mitochondrial proton-transporting ATP synthase complex
Mitochondrial protein complex
Mitochondrial membrane
Mitochondrial inner membrane
Mitochondrial envelope
Membrane protein complex
Membrane
Intracellular
Inner mitochondrial membrane protein complex
Cytoplasm
Cytochrome complex
Cellular anatomical entity
Alpha,alpha-trehalose-phosphate synthase complex (UDP-forming)
Strength
20
40
60
FDR
0.04
0.03
0.02
0.01
E
Pathway Enrichment Analysis
Small ribosomal subunit
Small nucleolar ribonucleoprotein complex
Ribosome
Ribosomal subunit
Ribonucleoprotein complex
Protein-containing complex
Preribosome, large subunit precursor
Preribosome
Nucleolus
Intracellular organelle
Intracellular non-membrane-bounded organelle
Intracellular
Endoplasmic reticulum membrane
Cytosolic small ribosomal subunit
Cytosolic ribosome
Cytosolic large ribosomal subunit
Cytosol
Cytoplasmic stress granule
Cytoplasmic ribonucleoprotein granule
Cytoplasm
Cellular anatomical entity
Box H/ACA snoRNP complex
Box C/D snoRNP complex
90S preribosome
Strength
25
50
75
100
FDR
0.04
0.03
0.02
0.01
F
Pathway Enrichment Analysis
Intracellular
Cytosol
Cytoplasm
Cellular anatomical entity
FDR
0.004
0.003
0.002
0.001
Strength
6.5
7.0
7.5
8.0
8.5

Figure S2

Figure S3. RT-qPCR analysis of RP genes. A and **B** are the mean and SD of three independent biological replicates with the RP gene-specific signal normalized to the internal reference control gene *SPT15*. Significance was determined by two-way ANOVA. *-$p < 0.05$ or greater. Data are plotted on a Log_{10} scale.

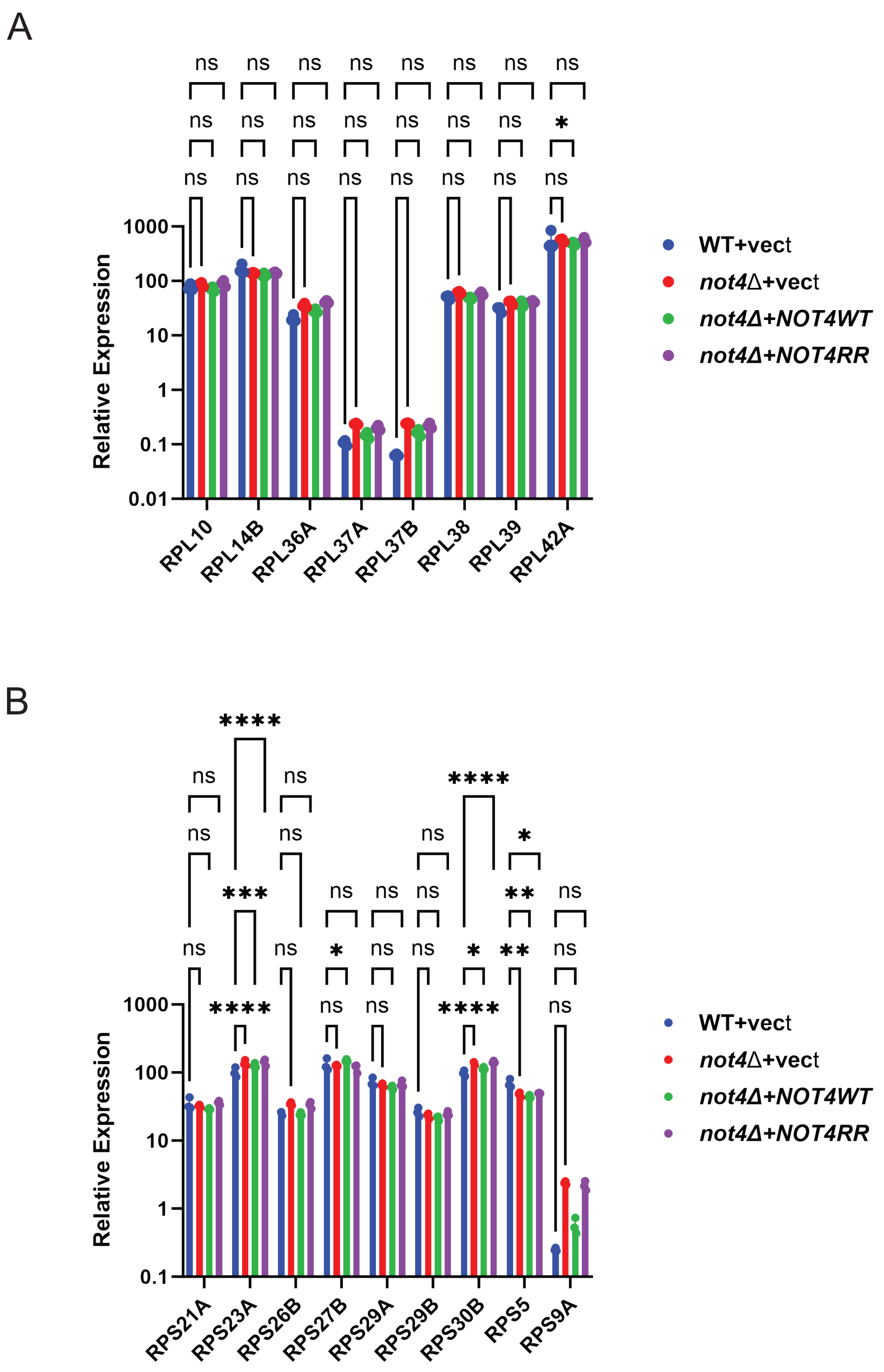
A
ns ns ns ns ns ns ns ns
ns ns ns ns ns ns ns *
ns ns ns ns ns ns ns ns
1000
100
10
1
0.1
0.01
Relative Expression
RPL10
RPL14B
RPL36A
RPL37A
RPL37B
RPL38
RPL39
RPL42A
WT+vect
not4Δ+vect
not4Δ+NOT4WT
not4Δ+NOT4RR
B
****
ns ns
****
ns ns ns *
*** ns ns ns ** ns
ns ns * ns ns * ** ns
**** ns ns **** ns
1000
100
10
1
0.1
Relative Expression
RPS21A
RPS23A
RPS26B
RPS27B
RPS29A
RPS29B
RPS30B
RPS5
RPS9A
WT+vect
not4Δ+vect
not4Δ+NOT4WT
not4Δ+NOT4RR

Figure S3

Figure S4. Proteomic quantification of the known Not4 substrates Rps7a, Egd1, and Egd1. The mean and SD of the normalized protein abundance for each indicated protein is plotted. The fold change relative to the WT + vector control was less than 1.5 for each protein, and the significance of the individual replicates between each condition was analyzed by one way ANOVA. *-$p<0.05$.

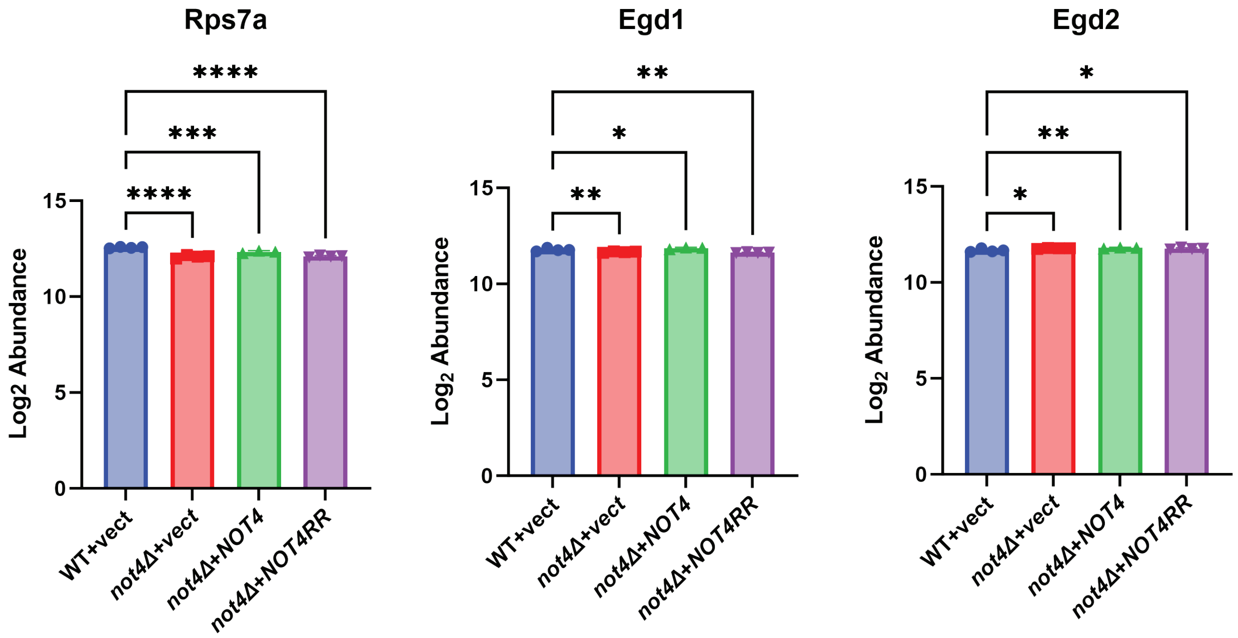
Rps7a
Egd1
Egd2
Log2 Abundance
Log2 Abundance
Log2 Abundance
****
***
****
**
*
**
*
**
*
0
5
10
15
WT+vect
not4Δ+vect
not4Δ+NOT4
not4Δ+NOT4RR

Figure S4

Figure S5. STRING analysis of the individual Venn components. The proteins in each component of the Venn from **Figure 1** were analyzed via the STRING database using a 0.9 confidence setting with the unconnected nodes removed. The arrows align the remaining connected networks to their respective Venn section. Only those networks related to RP and Ribi function are labeled.

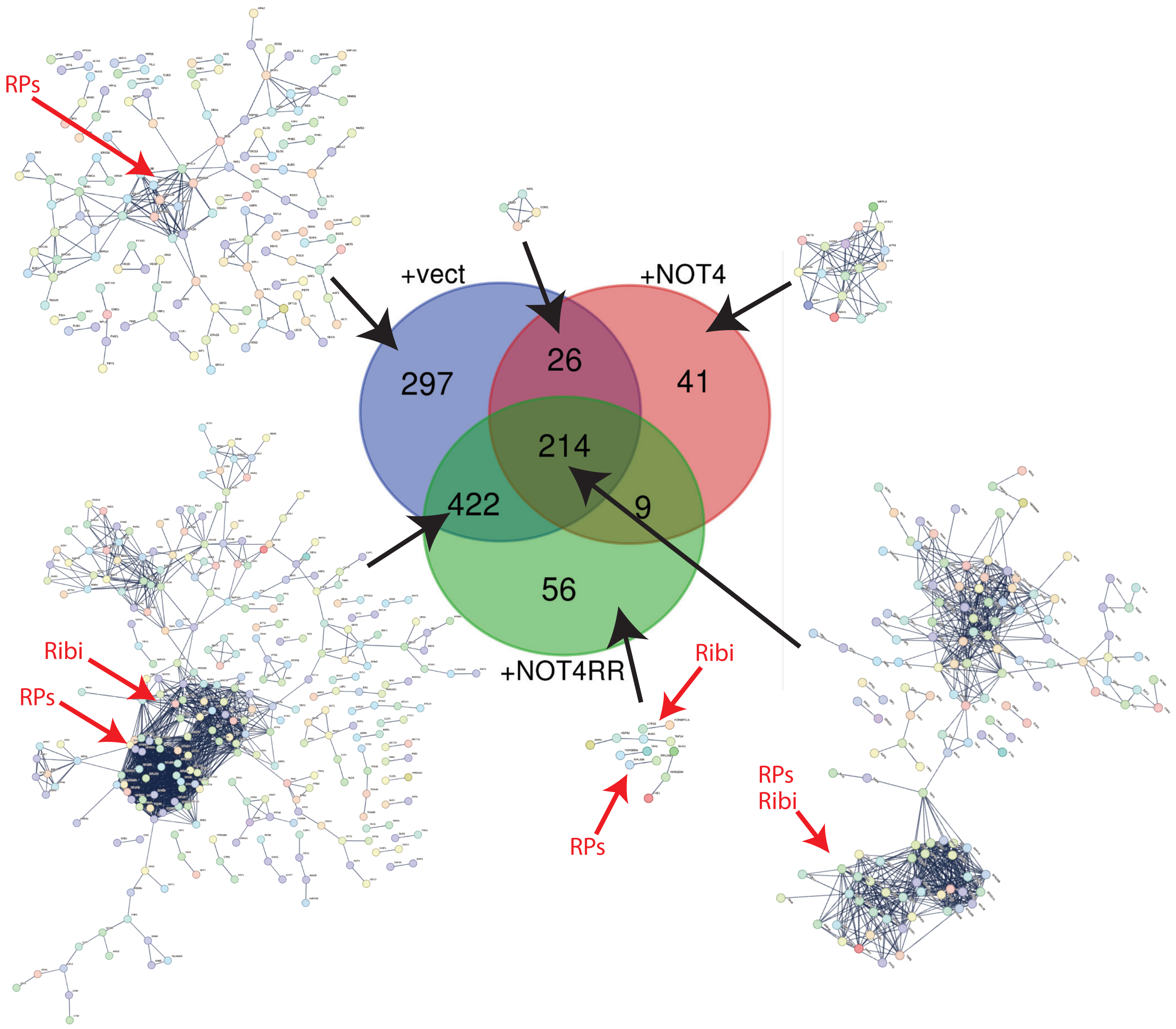

+vect
+NOT4
+NOT4RR
297
26
41
214
422
9
56
RPs
Ribi
RPs
Ribi
RPs
RPs
Ribi

Figure S5

Figure S6. Confocal microscopy of wild-type Rps9a-GFP. Log phase WT Rps9a-GFP cells stained with the vacuole-specific dye FM4-64 were analyzed by confocal microscopy for Rps9a-GFP distribution (**A**), vacuole localization (**B**), and their co-localization (**C**). Scale bar- 5 µm.

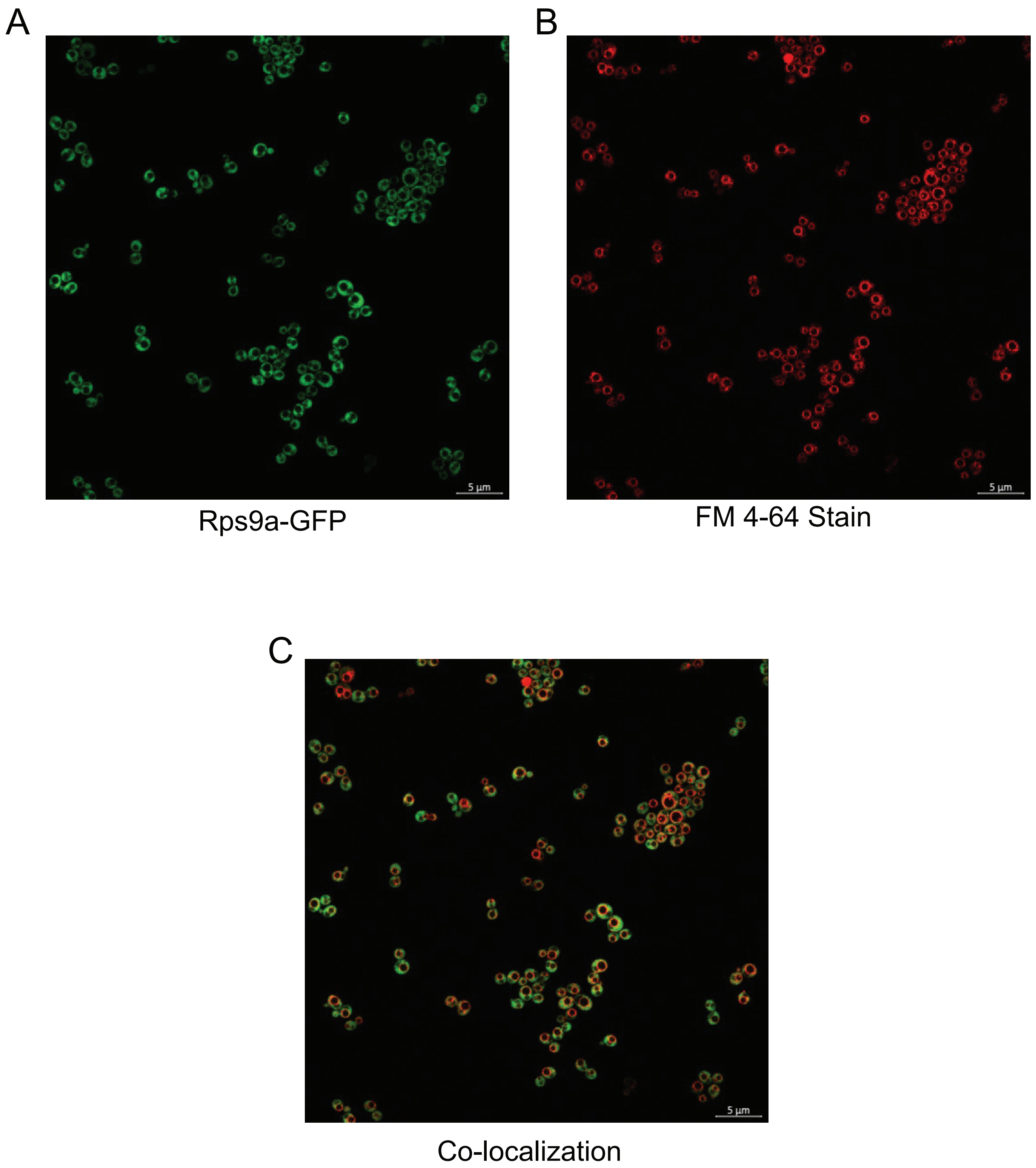

Figure S6

Figure S7. RT-qPCR analysis of *ATG* genes. **A** and **B** are the mean and SD of three independent biological replicates with the *ATG* gene-specific signal normalized to the internal reference control gene *SPT15*. Significance was determined by two-way ANOVA. ****-$p<0.0001$. Data are plotted on a Log_{10} scale.

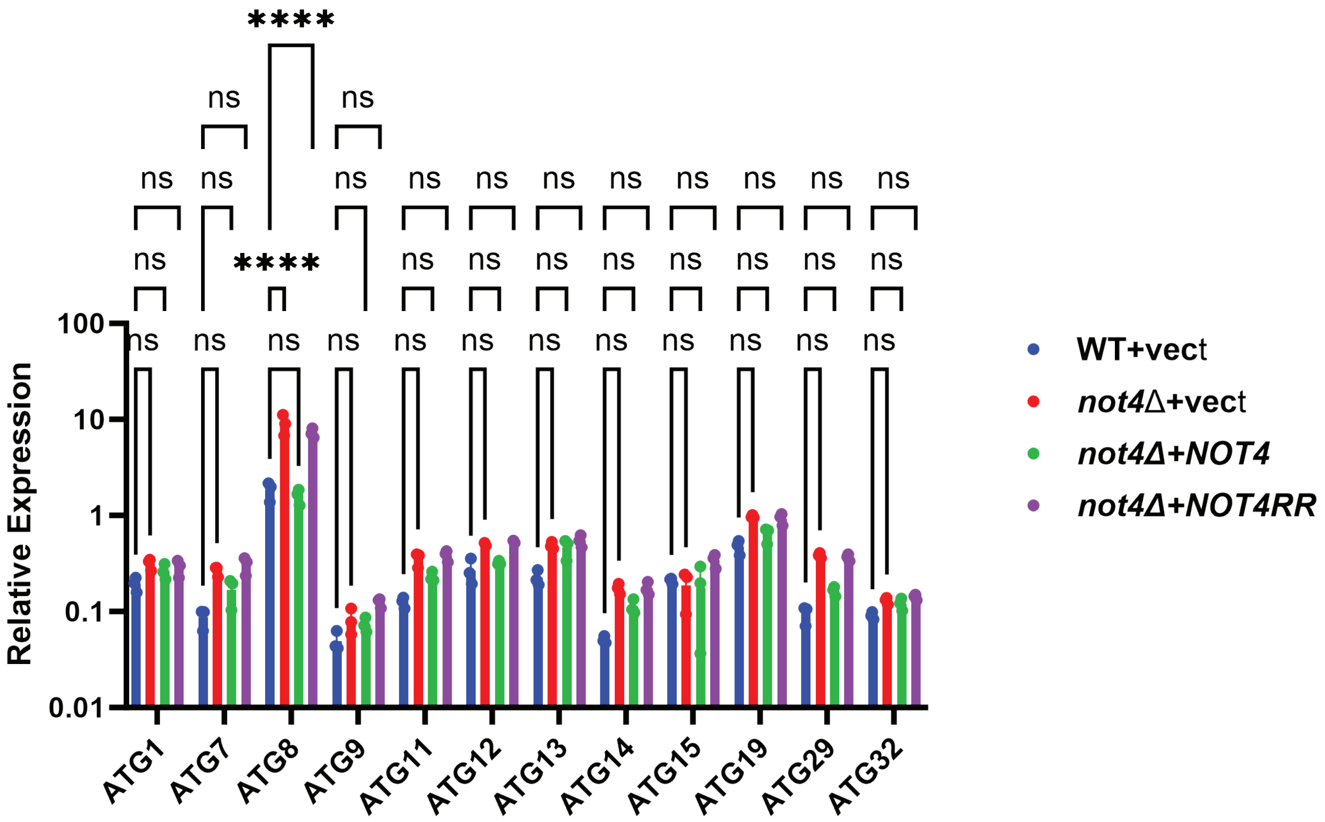
Relative Expression
100
10
1
0.1
0.01
ATG1
ATG7
ATG8
ATG9
ATG11
ATG12
ATG13
ATG14
ATG15
ATG19
ATG29
ATG32
ns
****
WT+vect
not4Δ+vect
not4Δ+NOT4
not4Δ+NOT4RR

Figure S7

Figure S8. Proteomic quantification of the autophagy factors Cue5, Nvj1, and Vps27. The mean and SD of the normalized protein abundance for each indicated protein is plotted. The fold increase relative to the WT + vector control for Cue5, Nvj1, and Vps27 in the *not4Δ* + vector and *not4Δ* + *NOT4RR* samples was 1.5 fold or higher. The significance of the individual replicates between each condition was analyzed by one way ANOVA. *-$p<0.05$.

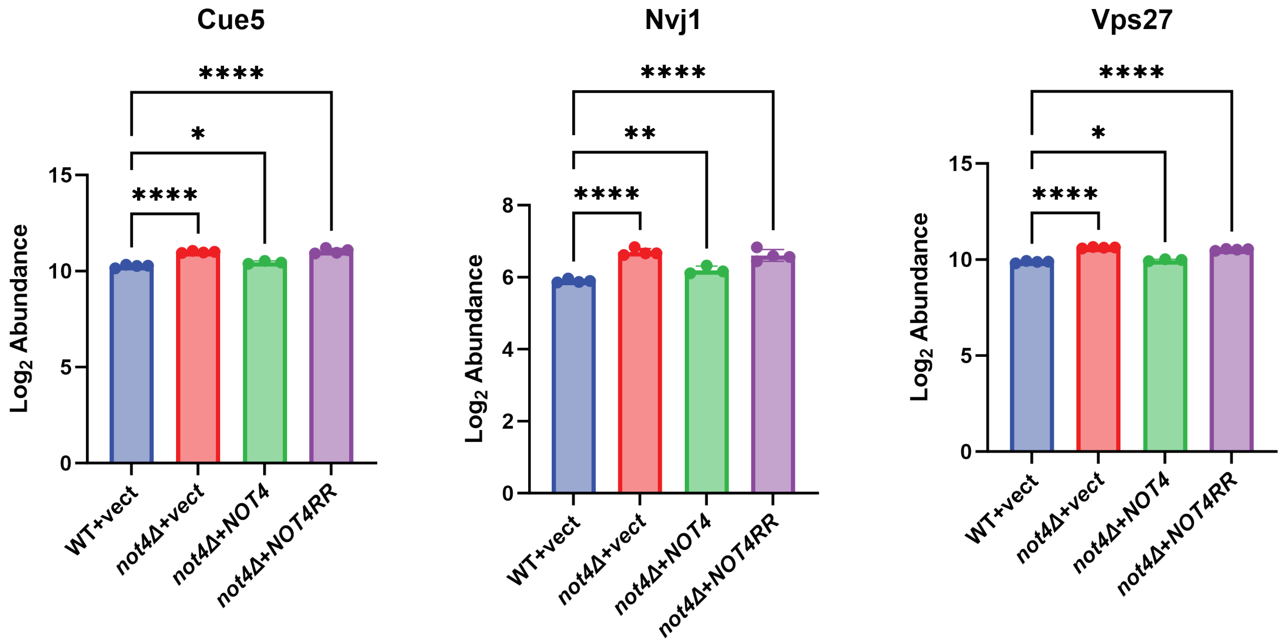

Cue5
Nvj1
Vps27
****
*
**
Log2 Abundance
WT+vect
not4Δ+vect
not4Δ+NOT4
not4Δ+NOT4RR

Figure S8

Figure S9. Proteomic quantification of the 60S ribophagy factors Ltn1, Ubp3, and Bre5. The mean and SD of the normalized protein abundance for each indicated protein is plotted. The fold change relative to the WT + vector control for Ltn1 and Ubp3 was less than 1.5 fold in all the experimental conditions. Bre5 expression was reduced in the *not4Δ* + vector (-1.96 fold) and it was reduced in the *not4Δ* + *NOT4RR* (-1.75 fold). The significance for the individual replicates between each condition was analyzed by one way ANOVA. *-$p<0.05$.

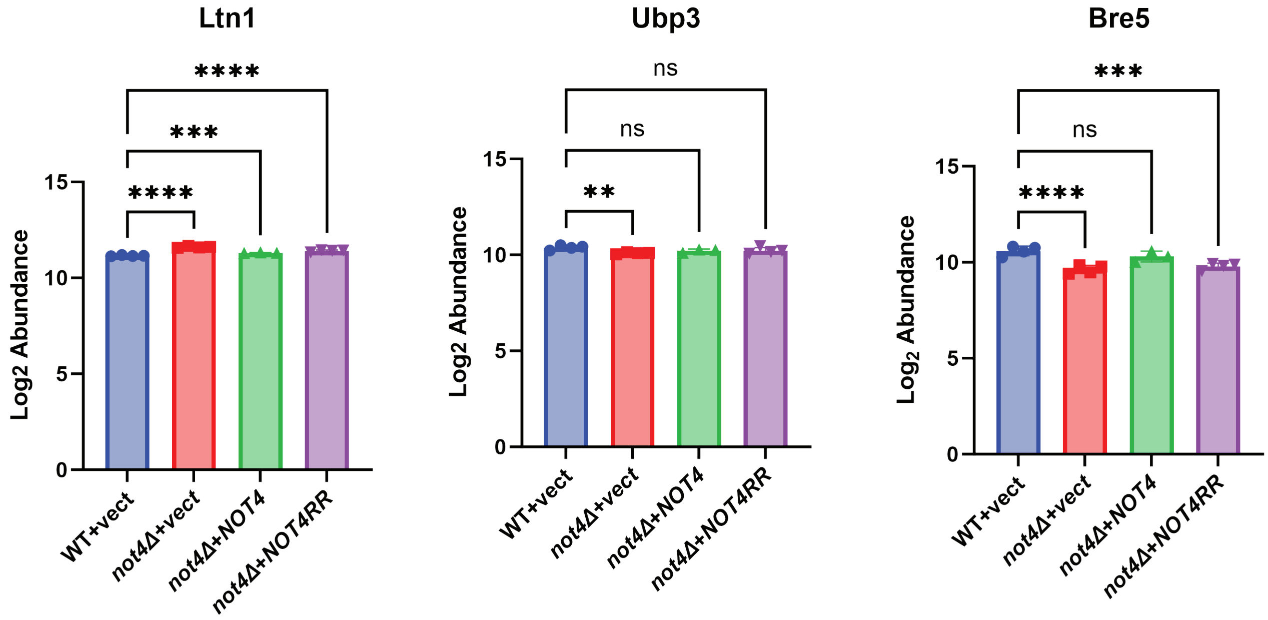

Ltn1
****
***
****
Log2 Abundance
15
10
5
0
WT+vect
not4Δ+vect
not4Δ+NOT4
not4Δ+NOT4RR
Ubp3
ns
ns
**
Log2 Abundance
15
10
5
0
WT+vect
not4Δ+vect
not4Δ+NOT4
not4Δ+NOT4RR
Bre5
***
ns
****
Log2 Abundance
15
10
5
0
WT+vect
not4Δ+vect
not4Δ+NOT4
not4Δ+NOT4RR

Figure S9
