# Supplementary material for: Ccr4-not ubiquitin ligase signaling regulates ribosomal protein homeostasis and inhibits 40S ribosomal autophagy
Source: J Biol Chem. 2024 Jul 16;300(8):107582. doi: 10.1016/j.jbc.2024.107582 (PMC11357857; doi:10.1016/j.jbc.2024.107582)
Supplement: Table S1 [file mmc5.docx]

**Table S1. Yeast strains.**

| **Strain** | **Description** | **Reference** |
| --- | --- | --- |
| BY4741 | *MATa his3-D1 leu2-D0 met15-D0 ura3-D0* | Dharmacon/  Open Biosystems |
| *ccr4Δ* | *MATa his3-D1 leu2-D0 met15-D0 ura3-D0 ccr4D::K KanMX* | Dharmacon/  Open Biosystems |
| *not4Δ* | *MATa his3-D1 leu2-D0 met15-D0 ura3-D0 not4D::KanMX* | Dharmacon/  Open Biosystems |
| YNL970 | *MATa his3-D1 leu2-D0 met15-D0 ura3-D0 RPS30B-EGFP::KanMX* | This study |
| YNL988 | *MATa his3-D1 leu2-D0 met15-D0 ura3-D0 RPS30B-EGFP::KanMX not4Δ:natNT2* | This study |
| YNL992 | *MATa his3-D1 leu2-D0 met15-D0 ura3-D0 RPS9A-EGFP::KanMX* | This study |
| YNL994 | *MATa his3-D1 leu2-D0 met15-D0 ura3-D0 RPS9A-EGFP::KanMX not4D::natNT2* | This study |
| YNL995 | *MATa his3-D1 leu2-D0 met15-D0 ura3-D0 RPL36A-EGFP::KanMX* | This study |
| YNL997 | *MATa his3-D1 leu2-D0 met15-D0 ura3-D0 RPL36A-EGFP::KanMX not4D::natNT2* | This study |
| YNL998 | *MATa his3-D1 leu2-D0 met15-D0 ura3-D0 RPS9A-EGFP::KanMX ccr4D::natNT2* | This study |
| YNL1000 | *MATa his3-D1 leu2-D0 met15-D0 ura3-D0 RPS9A-EGFP::KanMX ccr4D::natNT2* | This study |
| YNL1001 | *MATa his3-D1 leu2-D0 met15-D0 ura3-D0 RPS9A-EGFP::KanMX pdr5D::* *HphNT1* | This study |
| YNL1003 | *MATa his3-D1 leu2-D0 met15-D0 ura3-D0 RPS9A-EGFP::KanMX atg7D::* *HphNT1* | This study |
| YNL1009 | *MATa his3-D1 leu2-D0 met15-D0 ura3-D0 RPS9A-EGFP::KanMX atg7D::* *HphNT1 not4D::natNT2* | This study |
| YNL1005 | *MATa his3-D1 leu2-D0 met15-D0 ura3-D0 RPS9A-EGFP::KanMX pdr5D::* *HphNT1 not4D::natNT2* | This study |
| YNL1014 | *MATa his3-D1 leu2-D0 met15-D0 ura3-D0 RPS9A-EGFP::KanMX cue5D::* *HphNT1* | This study |
| YNL1018 | *MATa his3-D1 leu2-D0 met15-D0 ura3-D0 RPS9A-EGFP::KanMX nhx1D::HphNT1* | This study |
| YNL1020 | *MATa his3-D1 leu2-D0 met15-D0 ura3-D0 RPS9A-EGFP::KanMX nvj1D::* *HphNT1* | This study |
| YNL1022 | *MATa his3-D1 leu2-D0 met15-D0 ura3-D0 RPS9A-EGFP::KanMX vps27D::* *HphNT1* | This study |
| YNL1024 | *MATa his3-D1 leu2-D0 met15-D0 ura3-D0 RPS9A-EGFP::KanMX cue5D::* *HphNT1 not4D::natNT2* | This study |
| YNL1025 | *MATa his3-D1 leu2-D0 met15-D0 ura3-D0 RPS9A-EGFP::KanMX nhx1D::HphNT1 not4D::natNT2* | This study |
| YNL1027 | *MATa his3-D1 leu2-D0 met15-D0 ura3-D0 RPS9A-EGFP::KanMX nvj1D::* *HphNT1 not4D::natNT2* | This study |
| YNL1029 | *MATa his3-D1 leu2-D0 met15-D0 ura3-D0 RPS9A-EGFP::KanMX vps27D::* *HphNT1 not4D::natNT2* | This study |
| YNL1075 | *MATa his3-D200 leu2-3,112 ura3-52 lys2-801 trp1-1gal2 RPS9A-EGFP::KANMX* | This study |
| YNL1077 | *MATa his3-D200 leu2-3,112 ura3-52 lys2-801 trp1-1 ubc4-D1::HIS3 ubc5-D1::LEU2 RPS9A-EGFP::KANMX* | This study |
| YNL1081 | *MATa his3-D1 leu2-D0 met15-D0 ura3-D0 SRP40-EGFP::KanMX* | This study |
| YNL1083 | *MATa his3-D1 leu2-D0 met15-D0 ura3-D0 SRP40-EGFP::KanMX not4Δ:natNT2* | This study |
| YNL1085 | *MATa his3-D1 leu2-D0 met15-D0 ura3-D0 PRP11-EGFP::KanMX* | This study |
| YNL1087 | *MATa his3-D1 leu2-D0 met15-D0 ura3-D0 PRP11-EGFP::KanMX not4Δ:natNT2* | This study |
