# Supplementary material for: Ccr4-not ubiquitin ligase signaling regulates ribosomal protein homeostasis and inhibits 40S ribosomal autophagy
Source: J Biol Chem. 2024 Jul 16;300(8):107582. doi: 10.1016/j.jbc.2024.107582 (PMC11357857; doi:10.1016/j.jbc.2024.107582)
Supplement: Table S2 [file mmc6.docx]

**Table S2. Yeast Plasmids.**

| **Plasmid** | **Description** | **Reference** |
| --- | --- | --- |
| p416ADH | *AmpR CEN6/ARSH4 URA3 ADH1prom; CYC1term* | (1) |
| pRS416 | *AmpR CEN6/ARSH4 URA3* | (2) |
| pRS415 | *AmpR CEN6/ARSH4 LEU2* | (2) |
| pNOT4 | *AmpR CEN6/ARSH4 URA3 ADH1prom-NOT4-FLAG; CYC1term* | (3) |
| pNOT4RR | *AmpR CEN6/ARSH4 URA3 ADH1 prom-NOT4 I64A/G167A//F202A/C244A-FLAG; CYC1 term* | (3) |
| pNOT4ORF | *pR416; NOT4ORF (300 bp of promoter and 100 bp downstream of translational stop)* | This study |
| pPL156 | pPL132, *HA3-TOR1* I1954V | (4) |
| pRS416 GFP-ATG8 | *pRS416; ATG8prom-GFP-ATG8* | (5) |
| pPL156 | *LEU2, CEN/ARS HA3-TOR1^I1954V^* | (4) |

**References**

1. Mumberg, D., Muller, R. and Funk, M. (1995) Yeast vectors for the controlled expression of heterologous proteins in different genetic backgrounds. *Gene*, **156**, 119-122.

2. Sikorski, R.S. and Hieter, P. (1989) A system of shuttle vectors and yeast host strains designed for efficient manipulation of DNA in Saccharomyces cerevisiae. *Genetics*, **122**, 19-27.

3. Chen, H., Sirupangi, T., Wu, Z.H., Johnson, D.L. and Laribee, R.N. (2018) The conserved RNA recognition motif and C3H1 domain of the Not4 ubiquitin ligase regulate in vivo ligase function. *Sci Rep*, **8**, 8163.

4. Reinke, A., Chen, J.C., Aronova, S. and Powers, T. (2006) Caffeine targets TOR complex I and provides evidence for a regulatory link between the FRB and kinase domains of Tor1p. *J Biol Chem*, **281**, 31616-31626.

5. Guan, J., Stromhaug, P.E., George, M.D., Habibzadegah-Tari, P., Bevan, A., Dunn, W.A., Jr. and Klionsky, D.J. (2001) Cvt18/Gsa12 is required for cytoplasm-to-vacuole transport, pexophagy, and autophagy in Saccharomyces cerevisiae and Pichia pastoris. *Mol Biol Cell*, **12**, 3821-3838.
